# Enhancers regulate genes linked to severe and mild childhood asthma

**DOI:** 10.1016/j.heliyon.2024.e34386

**Published:** 2024-07-09

**Authors:** Tahmina Akhter, Enrichetta Mileti, Maura M. Kere, Johan Kolmert, Jon R. Konradsen, Gunilla Hedlin, Erik Melén, Carsten O. Daub

**Affiliations:** a Department of Biosciences and Nutrition, Karolinska Institutet, 141 83, Stockholm, Sweden; b Department of Clinical Science and Education, Södersjukhuset, Karolinska Institutet, 118 83, Stockholm, Sweden; c Institute of Environmental Medicine, Karolinska Institutet, 171 77, Stockholm, Sweden; d Department of Women's and Children's Health, Karolinska Institutet, 171 77, Stockholm, Sweden; e Science for Life Laboratory, Stockholm, Sweden

**Keywords:** Enhancer, Gene regulation, Gene expression, Childhood asthma, Severe asthma, Peripheral blood leukocytes

## Abstract

**Background:**

Children with severe asthma suffer from recurrent symptoms and impaired quality of life despite advanced treatment. Underlying causes of severe asthma are not completely understood, although genetic mechanisms are known to be important.

**Objective:**

The aim of this study was to identify gene regulatory enhancers in leukocytes, to describe the role of these enhancers in regulating genes related to severe and mild asthma in children, and to identify known asthma-related SNPs situated in proximity to enhancers.

**Methods:**

Gene enhancers were identified and expression of enhancers and genes were measured by Cap Analysis Gene Expression (CAGE) data from peripheral blood leukocytes from children with severe asthma (n = 13), mild asthma (n = 15), and age-matched controls (n = 9).

**Results:**

From a comprehensive set of 8,289 identified enhancers, we further defined a robust sub-set of the high-confidence and most highly expressed 4,738 enhancers. Known single nucleotide polymorphisms, SNPs, related to asthma coincided with enhancers in general as well as with specific enhancer-gene interactions. Blocks of enhancer clusters were associated with genes including TGF-beta, PPAR and IL-11 signaling as well as genes related to vitamin A and D metabolism. A signature of 91 enhancers distinguished between children with severe and mild asthma as well as controls.

**Conclusions:**

Gene regulatory enhancers were identified in leukocytes with potential roles related to severe and mild asthma in children. Enhancers hosting known SNPs give the opportunity to formulate mechanistic hypotheses about the functions of these SNPs.

## 1 Introduction

Asthma is a chronic inflammatory airway disease associated with multiple predisposing genetic and environmental factors [1]. Asthma is associated with symptoms like coughing and dyspnea, and is characterized by reversible airflow obstruction, airway inflammation and bronchial hyperreactivity [2].

There is a significant hereditary component behind asthma, and asthma has been studied through both genome-wide association and gene expression studies. Specifically for childhood-onset asthma, at least 24 studies have reported 492 genome-wide significant associations [3]. Gene expression refers to the formation of functional RNA based on the DNA sequence in a cell, and transcriptional differences can be evaluated between different types of asthma. In general, gene expression changes in blood cells mirror systemic immune responses. A gene expression study in the European U-BIOPRED cohort showed that differential expression between severe asthmatics and non-asthmatics was related to an increase in chemotaxis and migration of blood leukocytes, exemplifying the immunological changes behind asthma [4]. Some of these immune-related expression signatures appear to overlap between asthma and other allergic conditions such as rhinitis and eczema [5].

Gene expression is regulated by transcription factor (TF) proteins binding to gene promoter regions. TF proteins also bind to enhancer regions in the genome that are distant from the genes they regulate, but the spatial configuration of the chromatin facilitates the proximity of these TF-bound enhancer regions to gene promoters. This way, enhancer regions can regulate the expression of proximal genes but also of genes with distances of tens to hundreds of kilobases, reviewed in [6]. Various strategies have been employed to identify enhancers in genomic regions with open chromatin or with characteristic sets of histone modifications including H3K4Me1 and H3K27Ac. Actively expressed enhancers were found by profiling enhancer RNA (eRNA) with Cap Analysis Gene Expression (CAGE) RNA sequencing data [7]. CAGE technology measures 5′-ends of protein coding and non-coding transcript starting sites (TSS) as so-called tag clusters (TCs) where TSSs of different transcript isoforms of one gene can be characterized by several CAGE TCs.

More than 60 gene loci were described that might contribute to risk of asthma disease [8]. At the same time, most genomic variants found in genome-wide association studies (GWAS) are located in non-protein coding genomic regions making it challenging to associate the variants to genes and consequently allow for functional hypotheses for the variants [9]. Here, enhancer regions were shown to coincide with disease-associated SNPs more than would be expected by chance [7].

H3K4Me2 ChIP-Seq data of circulating T helper (Th) cells from 12 adult asthma and 12 adult healthy controls was used to identify enhancers and to show that Th2 cell-specific enhancers were highly enriched for asthma-associated SNPs [10]. H3K27ac ChIP-Seq data in bronchial epithelial cells (BECs) from four adults with asthma and three healthy controls described near significant enrichment for asthma SNPs in super enhancers [11].

The aim of this study was to identify enhancers based on eRNA from CAGE data, to find enhancer signatures related to severe or mild asthma, and to establish potential regulatory interactions between asthma-relevant enhancers and genes. For this, we here used our previously published CAGE RNA sequencing dataset of peripheral blood leukocytes from asthmatic children [12] recruited to the Swedish Search study, a national multicenter cross-sectional study [13,14]. This dataset is based on children aged 6–18 years diagnosed with severe asthma (SA, n = 13), mild asthma (MA, n = 15), and age-matched healthy controls (CTRL, n = 9). The children with severe asthma displayed increased blood neutrophil counts. A previous study in the Swedish SEARCH cohort identified differential gene expression patterns in severe and mild childhood-onset asthma which remained but were less pronounced when the blood neutrophil levels were included into the modeling [12,15]. Furthermore, aimed to identify known asthma-related SNPs situated in proximity to these enhancers.

## 2 Methods

### 2.1 Human samples and cohort

We used the CAGE data [12] based on blood samples from the Swedish Search Study [13] comprising mild (n = 15) and severe (n = 13) asthmatic children as well as healthy controls (n = 9). In brief, clinical data was collected through questionnaires, blood samples were acquired, and lung function was assessed by spirometry and methacholine provocation. A comprehensive description of the Swedish Search cohort is provided in [13] and the clinical characteristics of the children our CAGE data is based on is provided in our earlier publication [12]. SA refers to children who, despite level 4 medication, had unsatisfactory asthma control according to Global Initiative for Asthma (GINA) Strategy for Asthma Management and Prevention 2008, and used a high dose of inhaled corticosteroids (ICS) in combination with leukotriene receptor antagonists (LTRA) and/or a long-acting beta2-agonist (LABA) [13,16].

### 2.2 Identification of promoter regions

The CAGE technology performs 5′-end transcript sequencing and identifies transcription start sites (TSS) genome-wide for all samples. We obtained the CAGE data mapped to the human genome (hg19) from [12]. The sequencing was performed using the same single molecule HeliScopeCAGE protocol and average sequencing depth as it was used for all FANTOM5 CAGE samples. The sequencing depth ranged from 2.5 M to 6.9 M reads per sample, with an average of 4.9 M mapped reads per sample (Table S0). CAGE TSSs (CTSS) were clustered using Paraclu [17] into 78,176 tag-clusters (TCs), which identify the promoter regions of these transcripts. Raw tag counts for all TCs in all samples were normalized by the total tag count per sample (tags per million, TPM). TCs were annotated with GeneID and RefSeq gene models (Ensembl Biomart, downloaded 2020/06) using BEDtools intersect command with standard settings (Tables S1 and S2). We defined a *comprehensive* set of 78,196 TCs with minimum expression of one TPM in at least one sample as well as a *robust* set of 40,273 TCs with at least 3 TPM in all samples (Fig. S1).

### 2.3 Differentially expressed genes and enhancers

Comparison between TCs and enhancers of the MA and SA groups as well as to the control group were performed using EdgeR version 3.28.0 [18] based on raw count values and using glm with a design matrix for three groups. Statistical significance criteria (FDR) are provided in the respective sections.

### 2.4 Pathway analysis

We performed the pathway analysis to establish the relationships between biological processes and genes using WikiPathways [19] (March 10, 2021: “Version 20210310 released”) as source of biological processes. Enrichr integrative web based tool [20] was used to compute the enrichment analysis of asthma specific gene sets. The pathways linked to asthma specific TCs, enhancers, and enhancers cluster are reported in Tables S4, S11, and S7 respectively.

### 2.5 Enhancer identification

We identified enhancer regions based on balanced bidirectionally transcribed loci following the script provided by [7] with directionality score abs(D) > 0.9, ± 250 bp flanking window and bi-directional TC pairs separated by at most 450bp. We excluded (masked) the TCs coinciding with ENSEMBL or Gencode annotation as well as the 1,000 remaining most highly expressed TCs. Following the CAGE enhancer criteria established earlier [21], we identified 8,289 *comprehensive* enhancer candidates with at least 2 tags in 1 sample as well as 4,738 *robust* enhancers with at least 2 tags each in 6 samples. Enhancer identification with additional sets of more strict criteria, for example 2 tags in each of the 27 samples resulting in 1,306 enhancers, are provided in Table S6.

### 2.6 Confirmation of enhancers by epigenetic datasets

Genomic regions hosting active enhancers are typically associated with open chromatin and histone configurations including H3K4me1 and H3K27ac. We assessed how the identified enhancers coincided with i) enhancers identified by others and ii) epigenomic marks (Table 1) using BEDtools intersect command with standard settings.

**Table 1:** Identified enhancers are supported by enhancer databases and epigenetic marks. The robust enhancers are a subset of the comprehensive enhancer set. The FANTOM5 consortium identified enhancers from the same type of CAGE data we employed here for enhancer identification. The first column describes the data sources of enhancers with the total numbers of enhancers identified in these respective projects in brackets. For comprehensive enhancers (column 2) and robust enhancers (column 3), we display in absolute numbers and in corresponding percent how many of our identified enhancers are supported by the other datasets. Numbers in brackets refer to the enhancers we describe as *novel*, which were not identified in FANTOM5.

	comprehensive (novel)	robust (novel)
Identified enhancers	8,289 (3,277; 39.5 %)	4,738 (1,439; 30.4 %)
Confirmation with other enhancer sources and epigenomic marks		
ENCODE proximal enhancers (145,452)	1,899; 23 % (795; 24 %)	1,166; 25 % (430; 30 %)
ENCODE distal enhancers (677,050)	6,970; 84 % (2,067; 63 %)	4,564; 96 % (1,087; 76 %)
VISTA enhancers (998)	18; 0.2 % (3; 0.1 %)	11; 0.2 % (3; 0.2 %)
ATAC-Seq (590,651)	7,185; 87 % (2,488; 76 %)	4,386; 93 % (1232; 86 %)
H3K27ac monocyte (38,532)	3,108; 38 % (876; 27 %)	2,273; 48 % (557; 39 %)
H3K4me1 monocyte (122,647)	5,147; 62 % (1,538; 47 %)	3358; 71 % (818; 57 %)

We employed enhancers from.

● FANTOM5 (human comprehensive enhancers phase 1 and 2 from all tissues and cell types) [22].
● ENCODE proximal enhancers (pELS) and distal enhancers (dELS) [23], downloaded on 2021-03-30 and converted to hg19 using Assembly Converter [24] tool. Tissues: Peripheral blood mononuclear cells
● Human VISTA enhancers [25] (downloaded, 2021-03-30). Tissues: blood vessels

Chromatin conformation epigenomic marks were obtained as follows.

● Open chromatin data measured by ATAC-seq [26]. Tissue: Monocytes
● Histone modification marks, H3K4me1 and H3K27ac measured by ChIP-Seq peaks for monocyte subpopulations [27], downloaded from Rehli's lab by using track hubs. Tissue: Monocytes

### 2.7 Genomic clusters of densely positioned enhancers

Following [7], we have identified genomic clusters of densely positioned enhancers as consecutive regions of enhancers from the comprehensive enhancers set if they were closer than 15 kb (BEDtools cluster command) to each other. We associated genes within genomic enhancer clusters by extending both margins of the enhancers cluster with ± 10 kbp (Fig. S9, Table S7). Throughout this article, we used the short version “genomic enhancer clusters".

### 2.8 SNPs from GWAS in enhancer regions

We obtained 5,751 unique GWAS lead SNPs from the GWAS Catalog (v1.0, downloaded 2021-01-19) using asthma-related traits (Table 2 and Table S10) from the following selected European populations: CEU (Utah Residents with Northern and Western European ancestry), TSI (Toscani in Italia), FIN (Finnish in Finland), and IBS (Iberian population in Spain). We applied a threshold coefficient of determination (R^2^) of 0.8. We mapped GRCh38 SNPs from the GWAS catalog to GRCh37 using Assembly Converter [24] tool covering the SNP regions. We obtained 10,819 asthma-related SNPs in LD to the lead SNPs using LDlink (version v3.0) [28]. We used the genomic enhancer cluster boundaries for WikiPathway [20,29].

**Table 2:** GWAS SNPs coincide with identified enhancers. Using annotation terms relevant for asthma (column 1), GWAS database lead SNPs (column 2) and corresponding LD associated SNPs (column 3) were obtained and compared by exact coincidence with comprehensive enhancers and within 1 kB up- or down-stream of the SNP.

Asthma associated SNPs	Lead SNPs	LD associated SNPs	Compare Lead SNPs to comprehensive enhancers (1 kB up- or down-stream)	Compare LD SNPs to comprehensive enhancers (1 kB up- or down-stream)
Childhood onset asthma	492	1,478	7 (21)	10 (69)
Asthma	1,707	3,891	12 (61)	26 (159)
COPD	974	2,391	1 (14)	7 (44)
Response to bronchodilator	2,524	2,391	2 (19)	7 (44)
Forced expiratory volume (FEV)	1,699	3,260	1 (8)	4 (51)
FEV/FEC ratio	2,891	4,983	4 (29)	14 (73)
Unique SNPs total	5,751	10,819	16 (106)	60 (381)

### 2.9 Enhancers regulate gene promoters

Promoter-enhancer linkage prediction was performed by CAGE expression correlation across samples (Fig. S13). All samples were included in TPM normalization. Interactions between enhancers and TCs were considered when a) the genomic distance between a comprehensive promoter and a robust enhancer region was≤2Mbp and b) the expression correlation was R ≥ 0.5, FDR was not considered. The R function cor.test was used to test for the significance for Pearson correlation and the p.adjust function was employed to compute the associated False Discovery Rate (FDR) using Bonferroni correction (“bonferroni").

### 2.10 Hi-C and TAD data

We obtained three publicly available Hi-C datasets (Table S13).

Dataset 1 contains Hi-C data from human Peripheral blood T cells (GSM3967114, GSM3967115, GSM3967116) [30].

Dataset 2 contains Hi-C data from the Feng Yue Lab at Northwestern University (http://yuelab.org/) from one human lung donor.

Further details are provided in Table S13.

Dataset 3 contains promoter capture Hi-C data from human monocytes, B cells, CD4^+^ cells, CD8^+^ sorted cells and neutrophils [31] (https://osf.io/u8tzp/).

## 3 Results

From the CAGE data we identified a *comprehensive* set of 15,252 genes (78,176 TCs) with corresponding expression for all children (full dataset). In addition, we defined a so-called *robust* set of most highly expressed (above an average expression across all samples of 3 TPM) 13,489 genes (40,273 TCs) (see supplementary methods, Fig. S1, Tables S1–S2). A flowchart of the conducted analysis workflow is provided in Fig. S2.

### 3.1 Gene expression in leukocytes linked to mild and severe asthma

**Fig. 1:**
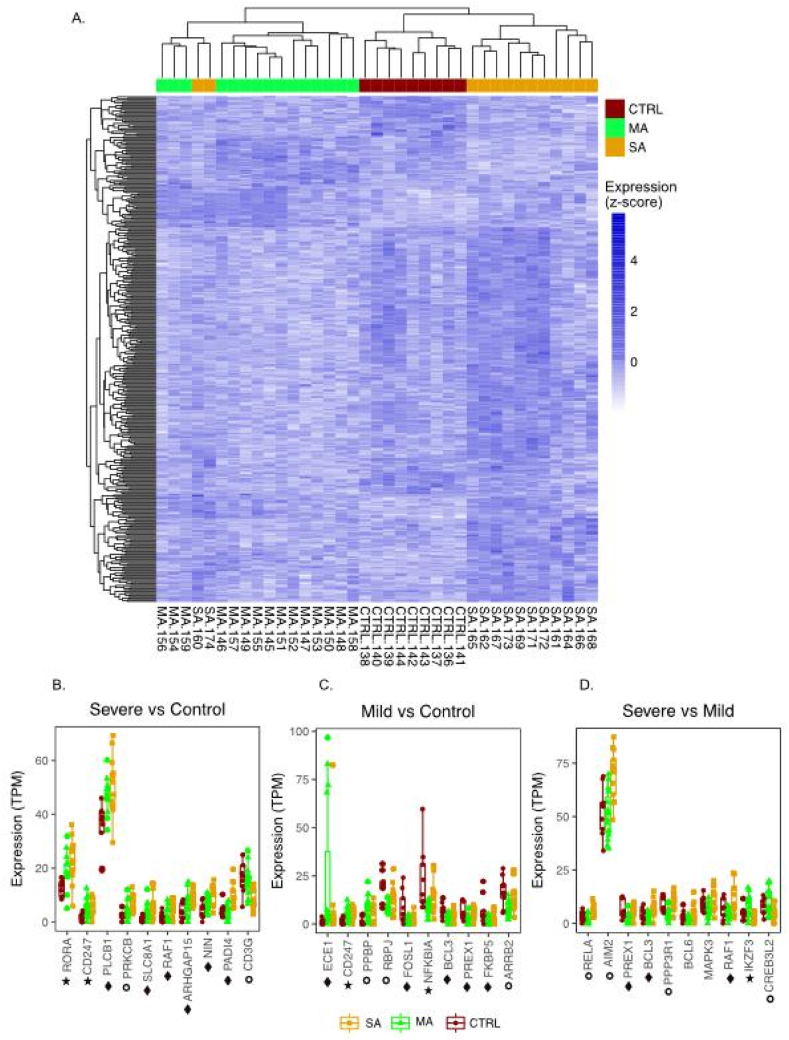
Genes linked to mild (MA) or severe asthma (SA). An expression signature of 321 genes distinguished between children with SA and MA and the control group (FDR 10 % for SA vs Ctrl and MA vs. Ctrl and 5 % for SA vs. MA) (A). Gene expression differences in leukocytes for ten selected asthma related genes in children with SA (B) or MA (C) compared to control as well as between the SA and MA groups (D). Star: genes with SNPs associated with the term “Childhood onset asthma”. Diamond: genes with SNPs associated with the term “asthma”. Circle: genes with publications related to the search term “asthma”.

The 3-way global expression comparison (using a global linear model, glm) identified a gene expression signature of 321 genes (381 TCs) distinguishing between blood samples from SA, MA and CTRL children (FDR 10 % for SA vs Ctrl and MA vs. Ctrl and 5 % for SA vs. MA) (Fig. 1A, Fig. S3, Table S3).

For blood leukocytes linked specifically to SA vs. control, we identified 73 genes (79 TCs, FDR <10 %) (Fig. 1B and Fig. S4). Overall, these genes are related to pathways associated with endothelin and chemokine as well as T-cell antigen receptor signaling (Table S4 with Gene Ontology results). For the MA vs. control comparison, we identified 90 genes (105 TCs, FDR <10 %) mirroring pathways involved in chemokine and T cell receptor signaling as well as Vascular Endothelial Growth Factor/Receptor 2 (VEGFA-VEGFR2) and Brain Derived Neurotrophic Factor (BDNF) signaling (Fig. 1C and Fig. S4). The contrast between SA and MA was more pronounced than the contrasts to the control group resulting in 225 genes (235 TCs) even when applying more strict significance criteria (FDR <5 %) (Fig. 1D and Fig. S4). Related pathways included interleukin (IL)-1, IL-3, IL-5 and IL-17 signaling as well as signaling for Transforming Growth factor (TGF)-beta, Tumor Necrosis Factor (TNF)-alpha, and BDNF. In addition, pathways involved in chemokine signaling and B cell receptor signaling were associated with some of the gene expression changes between the asthma groups.

Applying the global gene signature to an independent expression dataset from CD4^+^ and CD8^+^ T-cells from mild and severe asthmatics and controls [32] displayed a less pronounced but still recognizable separation between the three groups (Fig. S5).

### 3.2 Enhancers in leukocytes

Characteristic bi-directional expression patterns in CAGE data, so-called enhancer RNA (eRNA), can reveal the location of active gene enhancers in the genome. The expression of eRNAs have been used to estimate the enhancer activity level [7,33]. Employing earlier described methods [7], we identified a *comprehensive* enhancer set of 8,289 enhancers across the n = 37 blood derived leukocyte samples. We further defined a *robust* subset of 4,738 most highly active enhancers with expression above two CAGE tags each in at least six samples following the expression cut-off established by [21] (see Methods).

### 3.3 Enhancers are supported by characteristic epigenetic marks

In addition to eRNA expression, active enhancers are characterized by epigenetic marks such as by open chromatin (measured for example by ATAC-Seq) as well as by histone modification marks including H3K27 acetylation (H3K27ac) and H3K4 mono-methylation (H3K4me1) [34]. We found that the vast majority of our identified enhancers were supported by established epigenetic marks from publicly available epigenomic data derived from blood leukocytes (Methods) (Table 1). Furthermore, 60–70 % of our enhancers were also observed in the FANTOM5 genome annotation project, which employed CAGE data in a wide range of tissue samples for enhancer identification (Table 1). We refer to the 3,277 comprehensive and 1,439 robust enhancers we identified here beyond the FANTOM5 reference enhancer atlas as novel CAGE enhancers, or in short, as *novel* enhancers. The novel enhancers displayed overall levels of expression and genomic conservation comparable to the enhancers identified by the FANTOM5 project (Figs. S7 and S8) indicating similar levels of reliability of the identified enhancers. While none of the enhancer candidates coincided with enhancers identified in Th cells of adults with asthma by Seumois et al. some enhancers were previously identified in BECs of adults with asthma by McErlean et al. (Table S15).

### 3.4 Enhancers in the genome are organised in clusters

Coordinated regulation of the genes in the hox gene cluster during early embryonic development has at least in part been attributed to enhancers in close genomic proximity to the gene cluster [35]. Considering the positions of enhancers in the genome sequence, we identified genomic clusters of densely positioned enhancers [7], which we from here on referred to as genomic enhancer clusters. We identified 1,492 genomic enhancer clusters with the largest cluster hosting 12 enhancers and further 26 genomic enhancer clusters hosting more than eight enhancers each (Methods, Fig. S9, Table S7).

We evaluated whether genomic enhancer cluster regions would host genes with relevance to asthma by performing a pathway analysis. Genes around the largest 26 genomic enhancer clusters were associated with TGF-beta, PPAR and IL-11 signaling as well as vitamin A and D metabolism, to name a few (Table S8). For one genomic enhancer cluster on chromosome 2, we identified 12 genes associated with, for example, selective expression of chemokine receptors during polarization (Table S9).

### 3.5 Known genomic variants coincide with enhancers

Genome-wide association studies (GWAS) have identified genomic variants statistically related to a range of asthma phenotypes. These SNPs are, however, often located outside of the coding regions of genes making a mechanistic understanding challenging. Enhancers have been identified to coincide with a comparably large number of SNPs and the disease context of the SNP can coincide with the specific tissue the actual enhancer is transcribed in [7]. Some asthma related SNPs including their LD associated SNPs are indeed falling into the genomic regions of enhancers (exact or 1 kB up- or down-stream of the SNP) identified in this study (Table 2 for an overview and Table S10 for details including SNP rs numbers). For example, Strawberry Notch Homolog 2 (*SBNO2*) (Fig. S10A) and Solute Carrier Family 19 Member 1 (*SLC19A1*) (Fig. S10B) have been linked to asthma in previous GWA studies [36,37].

### 3.6 Enhancers linked to mild and severe asthma

**Fig. 2:**
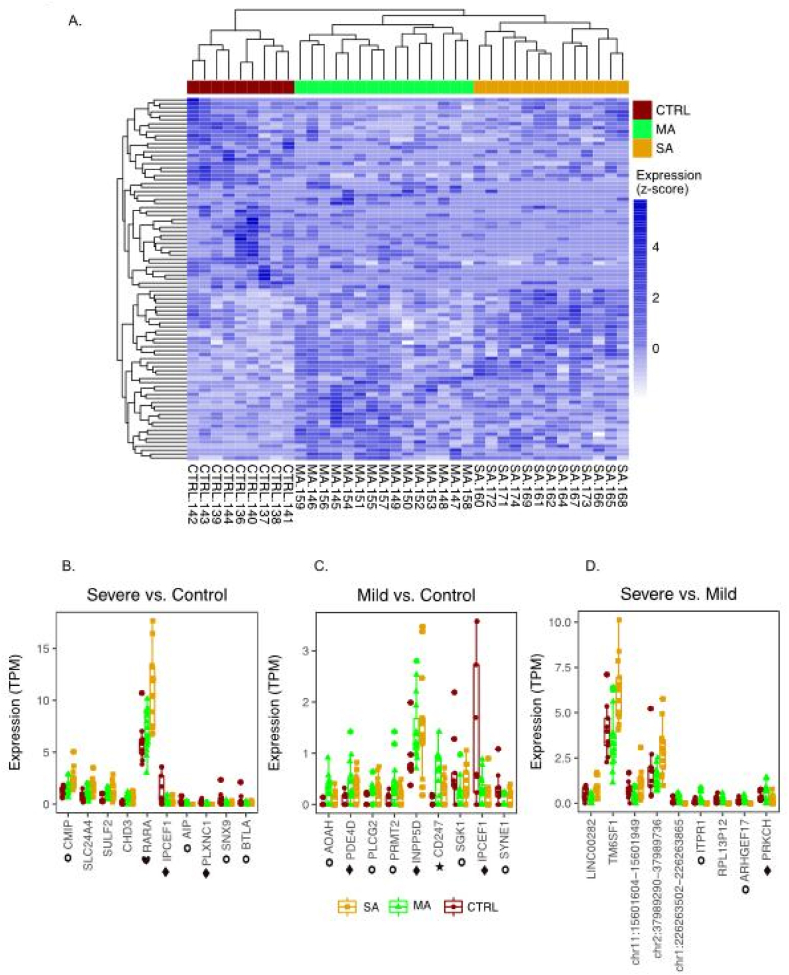
Enhancers linked to MA or SA. The activity of enhancers, measured by enhancer RNA (eRNA) expression in leukocytes, identified a signature of 91 enhancers that distinguished between MA, SA and control (FDR 30 % for SA vs MA and MA vs. Ctrl and 20 % for SA vs. Ctrl) (A). Expression of ten selected enhancers exemplified differences between severe (B) and mild asthma (C) compared to control as well as between the severe and mild asthma groups (D). Enhancers are named by their respective host genes when the enhancer is located in the intron of a gene. Star: genes with SNPs associated with the term “Childhood-onset asthma”. Diamond: genes with SNPs associated with the term “asthma”. Circle: genes with publications related to the search term “asthma”. Heart: genes with enhancers being part of a genomic enhancer cluster.

In analogy to obtaining the gene signature, we compared enhancer expression using a global linear model (glm) between the three groups of children resulting in a global enhancer signature composed of 91 enhancers distinguishing between SA, MA and controls (glm, FDR 30 % for SA vs MA and MA vs. Ctrl and 20 % for SA vs. Ctrl) (Fig. 2A, Fig. S11, Fig. S3 and Table S11). We identified 39 enhancers between SA and controls. One of these enhancers is possibly associated with the Retinoic Acid Receptor Alpha (*RARA*) gene, involved in vitamin A and carotenoid metabolism [38]. This enhancer was 2-fold up-regulated in SA (Fig. 2B and Fig. S11) and 1.4-fold (not significant) in MA (data not shown). Retinoids reportedly contribute to airway remodeling in asthmatic airways [39]. Enhancer expression analysis between MA and controls found 52 enhancers possibly related to pathways involved in immune functions like B cell and T cell antigen receptor signaling and Toll-Like Receptor (TLR)-4 signaling and tolerance (Fig. 2C and Fig. S11, Table S12). Between SA and MA, nine enhancers presumably coincided with genes linked to Resistin and Oncostatin M signaling as well as T cell antigen receptor signaling (Fig. 2D, Fig. S11, Fig. S12 and Table S12). We performed additionally a direct comparison between the SA and MA only, giving a signature of 91 enhancers (FDR< 30 %).

### 3.7 Enhancers possibly regulate expression of asthma genes

DNA is spatially organized in the chromatin in topologically associating domains (TADs). Within the TADs, enhancers and genes with up to several mega base pairs distance to each other in terms of genome coordinates can be in spatial proximity. Expression of enhancers and the genes they regulate has been shown to correlate [40]. For the 321 genes (Figs. 1A) and 91 enhancers (Fig. 2A) regulated in MA and SA, we identified 5,056 possibly interacting enhancer-gene pairs, where the enhancer and gene were in the same TAD within a distance of 2 mega base-pairs and exhibited an expression correlation of R ≥ 0.5, corresponding to FDR<0.125 (Methods) (Table 3, Fig. S13, Table S13). From the perspective of the asthma regulated enhancers, on the other hand, we found 33 supposably regulated genes with six of these genes reported in the asthma context (Table 4). We further obtained publicly available promoter capture Hi-C (PCHi-C) data from human monocytes, B cells, CD4^+^ cells, CD8^+^ sorted cells and neutrophils [31] to substantiate the gene-enhancer interaction candidates with experimental evidence.

**Table 3:** Enhancers interacting with genes. The number of enhancer-gene interactions are shown for comprehensive (column 2) or robust enhancers (column 3). Interacting enhancers can be supported by PCHi-C data obtained from different types of leukocytes, in genomic enhancer clusters, novel enhancers or in SNPs reported in the GWAS catalog. SNP numbers in brackets in column 1 indicate the total number of SNPs associated with a given term.

	With comprehensive enhancers	With robust enhancers
Enhancer-gene interactions (5,056)	1,636	1,466
PCHi-C (Monocytes) 4,754	1,505	1,356
PCHi-C (Neutrophils) 4,852	1,524	1,368
PCHi-C (CD4^+^) 4,820	1,522	1,366
PCHi-C (CD8^+^) 4,719	1,509	1,356
PCHi-C (B cells) 4,652	1,473	1,327
Enhancers in genomic enhancers clusters	667	620
Novel enhancers	520	423
GWAS SNPs related to *Childhood-onset asthma* (492)	1	1
GWAS SNPs related to *asthma* (1,707)	8	8
GWAS SNPs related to *COPD* (974)	1	1
GWAS SNPs related to *response to bronchodilator* (2,524)	2	2
GWAS SNPs related to *FEV/FEC ratio* (2,891)	6	6

**Table 4:** Enhancers regulate asthma related genes. Enhancers specific for severe or mild asthma or non-asthmatic controls (column 1) possibly regulate genes (column 2) including genes related to asthma (column 3). * Note: The sum of enhancers is larger than the number of unique enhancers since some enhancers are occurring in more than one group.

Asthma regulated comprehensive enhancers	regulating any gene	regulating asthma related genes
SA vs. CTRL (39)	12; (*LINC00937, RIN3, SLC24A4, C16orf54, CMIP, LOC101928947, SULF2, CD200R1, LOC100130476, TNFAIP3, TOX, RAPGEF1*)	2; (*SLC24A4, CMIP*)
MA vs. CTRL (52)	18; (*S100A9, ATP8B2, S100A12, MPZL1, TRAF3IP3, ERRFI1, CA6, IFITM3, SPATA33, DLGAP1, TTLL4, CXCR1, CTDSP1, SULF2, MARCKS, SGK1, SPIDR, PRKDC*)	4; (*APBA2, TTLL4, CD247, SLA2*)
SA vs. MA (9)	4; (*LBR, SOX6, LINC00282, LINC00211*)	0
Total unique enhancers* (91)	33	6

### 3.8 Examples of genes regulated by enhancers

Following a global analysis of genes, enhancers and gene-enhancer interactions related to genomic variation, we highlight specific genes with relevance for asthma.

#### 3.8.1 *RUNX3* is possibly regulated by three enhancers

*RUNX3* has been described in the context of pediatric asthma [41]

**Fig. 3:**
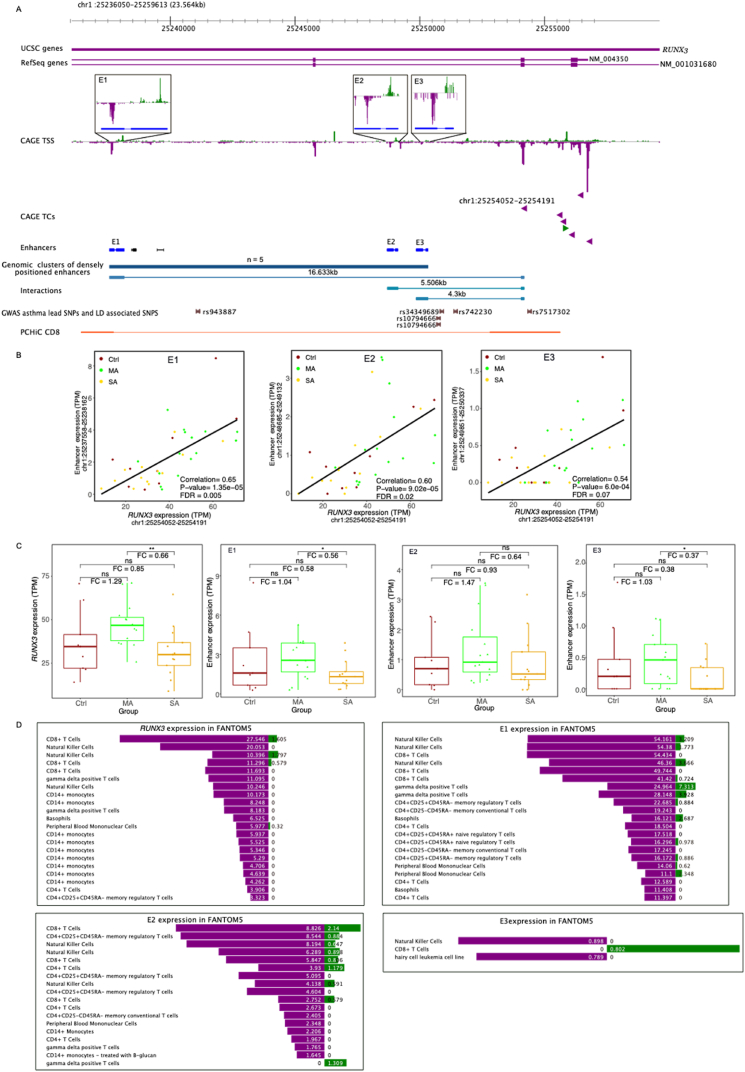
The regulation of the *RUNX3* gene potentially involves three enhancers located within its introns. (A) The panel displays gene models for UCSC genes and RefSeq genes. CAGE TSS shows the transcription starting sites (TSS) with expression in TPM: green (upwards) indicates expression data on the forward strand, while purple (downwards) represents the reverse strand. The zoom-in panels depict enhancer regions (E1, E2, E3). CAGE TCs are TSS grouped into tag-clusters (TCs), and enhancers are CAGE-defined for robust (blue) and comprehensive (black) enhancers. Genomic clusters of densely positioned enhancers are displayed in dark blue. Interactions show three potential enhancer-gene regulatory interactions, as detailed in panel (B). Asthma-related GWAS lead SNPs and LD-associated SNPs are shown in brown. PCHiC displays promoter capture HiC data from CD8 cells, with chromatin conformation data in CD8^+^ cells displayed in orange. (B) The expression of all three enhancers correlates with the expression of the *RUNX3* alternative promoter (Pearson correlation). (C) Both this promoter and the three enhancers were downregulated in SA compared to MA (FC: mean expression fold-change; Wilcoxon Rank Sum Test, *p < 0.05, **p < 0.005, non-significant for E2 but with the same direction of expression change). (D) According to the FANTOM5 database, the selected *RUNX3* promoter and the three enhancers are expressed in similar blood-related cell types.

And is expressed in CD8^+^ T-cells, CD14^+^ monocytes, and NK cells, amongst others (Fig. 3D). We observed reduced *RUNX3* expression in leukocytes from children with SA compared to MA (Fig. 3C). We found three robust enhancers in the introns of *RUNX3* (Fig. 3A) with reduced expression in children with SA compared to MA (Fig. 3C). According to FANTOM5 data, these enhancers were expressed in similar cell types as the *RUNX3* gene (Fig. 3D). The expression of one strong *RUNX3* promoter (chr1:25254052-25254191) correlated with the expression of the three enhancers (Fig. 3B) indicating that these enhancers might regulate the expression of this promoter. Publicly accessible chromatin conformation data from CD8^+^ cells supports the interaction with one of the enhancers (E1) [42]. Four asthma related SNPs are located in the *RUNX3* locus, three of these within a few kilobases distance from the enhancers (Fig. 3A), which might suggest a possible functional role for these enhancers. Additional examples of asthma related genes possibly regulated by the enhancers (*SBNO2, SLC19A1, CXCL1, VAV3, PTEN*) are provided in Fig. S10.

## 4 Discussion

As the first step, we re-defined CAGE tag-clusters from our previously published CAGE data of 28 children with asthma and 9 children without asthma as controls [12]. The new tag-cluster calling gave 40,273 robust and 78,176 comprehensive TCs compared to 221,465 TC used in [12]. The number of genes was marginally affected by this choice with 16,147 genes compared to 18,578 detected in [12].

We obtained a gene-expression signature of 321 genes (381 TCs) that distinguished the two asthma subtypes SA and MA from each other and from the control group. The gene expression signatures for two of the severely asthmatic children (SA160 and SA174) indicated that they might be wrongly classified into the mild asthmatic group. The diagnosis was clinically established and the details required to assess if these two individuals might be misclassified was not available under the ethical permits. The expression signature generalized to an independent microarray expression dataset of CD4^+^ and CD8^+^ T-cells [32].

Based on the characteristic bi-directional expression of enhancer RNA (eRNA), we identified a comprehensive set of 8,289 potential enhancers together with a subset of 4,738 highly expressed robust enhancers. The FANTOM5 project pioneered enhancer characterization with CAGE eRNA data. Enhancers found with CAGE data have previously been experimentally validated with higher success rate than enhancers found by the ENCODE project with RNA-Seq data or by epigenetic marks alone [7]. The FANTOM5 dataset comprises around ten samples derived from whole human blood and consequently, most of the enhancers we identified here in leukocytes, 60 % for comprehensive and 70 % for robust, have previously been described in FANTOM5. The dataset we employed here is composed of 37 samples making it more feasible to find expressed enhancers. The novel enhancers we found in addition to what was reported by FANTOM5 displayed an overall similar expression level (Fig. S6, Table S5) and openness of chromatin as measured by ATAC-Seq (Table 1) hinting at comparable levels of activity to FANTOM5 enhancers. The VISTA database hosts experimentally validated human and mouse enhancers across various tissues. Three of our novel CAGE enhancers were indeed reported in the VISTA database.

Identification of enhancers from CAGE data takes advantage of the emission of eRNA from enhancer loci. The amount of eRNA observed as bi-directional expression in CAGE data has been used as a proxy for the activity of the corresponding enhancer [7]. This necessity for an enhancer to be active in a sample in order to be observed in the corresponding CAGE data limits the ability to find enhancers with CAGE. Chromatin accessibility and conformation together with histone marks provide alternative and well established methods for enhancer identification, which might allow finding enhancers beyond the ones active at the given moment of sample taking.

A substantial number of the identified enhancers coincided with SNPs from GWAS related to lung diseases or lung function (Table 2). The enhancers can give clues as to how these SNPs might regulate gene expression possibly by influencing the efficacy of transcription factor protein binding to the enhancers. This, in turn, would affect the expression of the genes regulated by these enhancers. Around half of the enhancers containing SNPs were part of the genomic enhancer clusters, where the dense enhancer occurrence might suggest concerted regulatory programs for the genes in and around these genomic enhancer clusters. For 21 SNPs falling into enhancers out of a total of 492 SNPs associated with the term *Childhood onset asthma*, ten enhancers were organized in enhancer clusters.

The CAGE data allowed us to describe the identity, expression and activity of genes and enhancers. It remains challenging, however, to establish the specific regulatory relationship between enhancers and the genes they regulate. While enhancers often regulate the closest genes, there are reports of more complex regulatory relationships. In the absence of high resolution chromatin enhancer-promoter contact maps, we identified global topologically associating domains (TADs) from publicly available low-resolution chromatin conformation data. We considered possible interactions exclusively within these TADs and focused on the most proximal and thus most likely 5,056 specific interactions between enhancers and genes (Table 3). Of specific relevance were interactions where enhancers harbor known genomic variants (SNPs) related to lung diseases, which allows to formulate concrete hypotheses about the function of the respective SNPs.

The *RUNX3* gene locus was discussed in detail as one specific case of potential enhancer-promoter interaction. The gene was found to be expressed in many blood-related cell types according to the FANTOM5 and the GTEx expression atlases. Three enhancers were identified in the introns of the *RUNX3* gene. In the severe asthmatic children, the *RUNX3* gene, as well as these three enhancers, were downregulated as compared to children with mild asthma. As a consequence, expression of the three enhancers correlated with the expression of *RUNX3*. While the FANTOM5 database did not report expression of *RUNX3*, or any of the three enhancers in neutrophils, we can however not exclude that the observed increase in expression, at least in part, may be caused by increased blood neutrophil counts in the severe asthmatic children [13].

The H3K4Me2 histone mark was measured in CD4^+^ T-cells from peripheral blood in 12 asthmatic adults and 12 adult controls by Seumois et al. to identify 69,405 differentially enriched regions (DERs) as enhancers [10]. Gene expression was measured in all 24 samples using RNA-Seq. The 69,405 T-cell enhancers were associated with 7,600 T-cell expressed genes when enhancers and genes were not separated by any CTCF binding site (obtained from publicly accessible data). A total of 200 enhancer regions showed differential enrichment of H3K4me2 when comparing asthmatics to healthy controls. Furthermore, 52 out of 1,528 asthma related lead SNPs (from SNP database) were associated with enhancers.

There are several differences between our work and the work of Seumois et al. The data we used is based on leukocytes from children with severe or mild asthma and children without asthma, while Seumois et al. is based on adults with asthma and adult controls. The main difference is the identification of the enhancers, we took advantage of eRNA as measured with CAGE data while Seumois et al. employed H3K4Me2 histone modifications measured by ChIP-Seq. The most commonly used chromatin marks for enhancer identification are open chromatin (measured for example by ATAC-Seq or DNase-seq) in combination with H3K4Me1 and K3K27Ac histone modifications. The total number of H3K4Me2 defined enhancers is around ten times higher than the number of CAGE defined enhancers here. While the number of asthma related enhancers is similar between the two studies, 91 in our work and 200 in Seumois et al. the identified enhancers are different between the two studies.

McErlean et al. studied H3K27Ac histone modifications using ChIP-Seq in bronchial epithelial cells (BECs) from four adults with asthma and three healthy controls. 4,321 DERs were identified as enhancer candidates. Super enhancers were identified using the Rank Ordering of Super Enhancers algorithm. Compared to our study, the samples were obtained from BECs from the lung, which might be more directly relevant to asthma than blood samples. At the same time, the cohort was small with seven individuals in total. In the order of one hundred enhancers from our study were also identified as enhancers by McErlean et al. (Table S15).

Future directions could address the relevance of the tissue for gene signature and enhancer identification including BEC, blood or other tissues relevant for asthma. Regarding identification of enhancers, candidates can be found using epigenetic marks, by eRNA, with chromatin conformation measurements, or by combinations of these methods. Asthma has been described as an umbrella term for a number of distinct diseases, also called endotypes, with distinct underlying pathophysiological mechanisms. Addressing specific asthma endotypes might allow finding distinct enhancer or gene signatures. One additional major aspect is the generalization of observed asthma related enhancers signatures since the current reports considered small cohort sizes with up to 24 individuals. Improved experimental designs with independent discovery and validation cohorts will be important.

We employed publicly available CAGE sequencing data (Persson et al., 2015) from blood leukocytes from children in the Swedish SEARCH cohort (Konradsen et al., 2011) and identified expressed enhancers (eRNAs) at various levels of confidence and supported by publicly available epigenomic data. We further identified an enhancer expression signature distinguishing between SA, MA and control group children. Genomic variants (SNPs) from GWAS databases related to asthma coincided with identified enhancers. Potential regulatory associations between asthma-regulated enhancers and asthma-regulated genes were established based on co-expression and supported by publicly available chromatin conformation Hi-C data. The RUNX3 gene locus was selected to exemplify the possible interplay of enhancers, genes and SNPs.

## Data availability statement

All data and analysis scripts are available in the following Gitlab repository

https://gitlab.com/daub-lab/Enhancer_regulation_in_childhood_asthma

## CRediT authorship contribution statement

**Tahmina Akhter:** Writing – review & editing, Writing – original draft, Visualization, Investigation, Formal analysis. **Enrichetta Mileti:** Visualization, Formal analysis. **Maura M. Kere:** Formal analysis, Data curation. **Johan Kolmert:** Writing – original draft, Data curation. **Jon R. Konradsen:** Data curation. **Gunilla Hedlin:** Supervision. **Erik Melén:** Supervision. **Carsten O. Daub:** Writing – review & editing, Writing – original draft, Supervision, Project administration, Methodology, Investigation, Funding acquisition, Formal analysis, Conceptualization.

## Declaration of competing interest

The authors declare that they have no known competing financial interests or personal relationships that could have appeared to influence the work reported in this paper.
