# Supplementary material for: Enhancers regulate genes linked to severe and mild childhood asthma
Source: Heliyon. 2024 Jul 9;10(14):e34386. doi: 10.1016/j.heliyon.2024.e34386 (PMC11301366; doi:10.1016/j.heliyon.2024.e34386)
Supplement: Supplementary file 1 [file mmc1.pdf]

Enhancers regulate genes linked to severe and mild childhood asthma - Supplementary Material

Table of content

Availability of supplementary data **1**
Table S0 | Sequencing depth of publicly available CAGE dataset **2**
Figure S1 | TCs selection 4
Tables S1 and S2 | TCs annotation 4
Figure S2 | Flowchart: A computational workflow 6
Figure S3 | Genes, TCs and enhancers in expression signatures 7
Table S3 | Differentially expressed (DE) genes in TCs 8
Figure S4 | TCs linked to mild and severe asthma 8
Table S4 | WikiPathway analysis of DE TCs 8
Figure S5 | Test gene expression signatures in an independent dataset 9
Figure S6 and Table S5 | Compare TCs to FANTOM5 TCs 10
Table S6 | Classification of enhancers 12
Figure S7 | Identification of novel enhancers 13
Figure S8 | Conservation analysis of enhancer regions 14
Figure S9 | Identification of genomic enhancer cluster 15
Table S7 | Genomic clusters of densely positioned enhancers 15
Table S8 | WikiPathway analysis of genomic enhancers cluster 18
Table S9 | Genomic enhancers cluster on chromosome 2 19
Table S10 | GWAS asthma lead and LD associated SNPs (with gene associations) within enhancers region 19
Figure S10 | Genes are regulated by enhancers 20
Figure S11 | Enhancers linked to severe and mild asthma 29
Table S11 | Differentially expressed (DE) enhancers 29
Table S12 | WikiPathway analysis of DE enhancers 29
Figure S12 | Hierarchical clustering in severe and mild asthma 30
Figure S13 | Identify enhancer-TC interactions 31
Table S13 | Genes are regulated by enhancers 32
Table S14 | Enhancers are intersecting with genes 34
Figure S14 | Expression of RUNX3 across tissues 36
Table S15 | Enhancers overlap with McErlean et al.'s asthma enhancers 36
References **37**

Availability of supplementary data

All supplementary data and analysis scripts are available in the Gitlab repository under https://gitlab.com/daub-lab/Enhancer_regulation_in_childhood_asthma

Table S0 | Sequencing depth of publicly available CAGE dataset

We obtained publicly available CAGE data [1] based on blood samples from the Swedish Search Study [2] comprising mild (MA, n=15) and severe (SA, n=13) asthmatic children as well as healthy controls (n=9). Data were mapped to the human genome version hg19.

Sample name	class	Number mapped reads
CNhs12136	Control	6156525
CNhs12137	Control	5132952
CNhs12138	Control	4264527
CNhs12139	Control	6480347
CNhs12140	Control	5310856
CNhs12141	Control	5539723
CNhs12142	Control	5933314
CNhs12143	Control	7038891
CNhs12144	Control	5039763
CNhs12145	Mild asthma	3514438
CNhs12146	Mild asthma	5173740
CNhs12147	Mild asthma	3311560
CNhs12148	Mild asthma	3075962
CNhs12149	Mild asthma	4349014
CNhs12150	Mild asthma	4403091
CNhs12151	Mild asthma	6278138
CNhs12152	Mild asthma	5128145

CNhs12153	Mild asthma	4377877
CNhs12154	Mild asthma	4535643
CNhs12155	Mild asthma	5917332
CNhs12156	Mild asthma	3640736
CNhs12157	Mild asthma	4229594
CNhs12158	Mild asthma	4265629
CNhs12159	Mild asthma	6068663
CNhs12160	Severe asthma	6947378
CNhs12161	Severe asthma	3814947
CNhs12162	Severe asthma	6336892
CNhs12164	Severe asthma	2955848
CNhs12165	Severe asthma	4742283
CNhs12166	Severe asthma	2524324
CNhs12167	Severe asthma	4862531
CNhs12168	Severe asthma	2834504
CNhs12169	Severe asthma	6313759
CNhs12171	Severe asthma	5848329
CNhs12172	Severe asthma	5557468
CNhs12173	Severe asthma	5909519
CNhs12174	Severe asthma	4059435

Figure S1 | TCs selection

We identified 78,176 tag clusters (TCs) by using the PARACLU clustering method [3]. Then we normalized the raw expression counts as tags per million (TPM). Based on the total set of 78,176 TCs as comprehensive TCs, a subset of 40,273 TCs were defined as robust TCs after applying an expression cut off 3 TPM per sample [4].

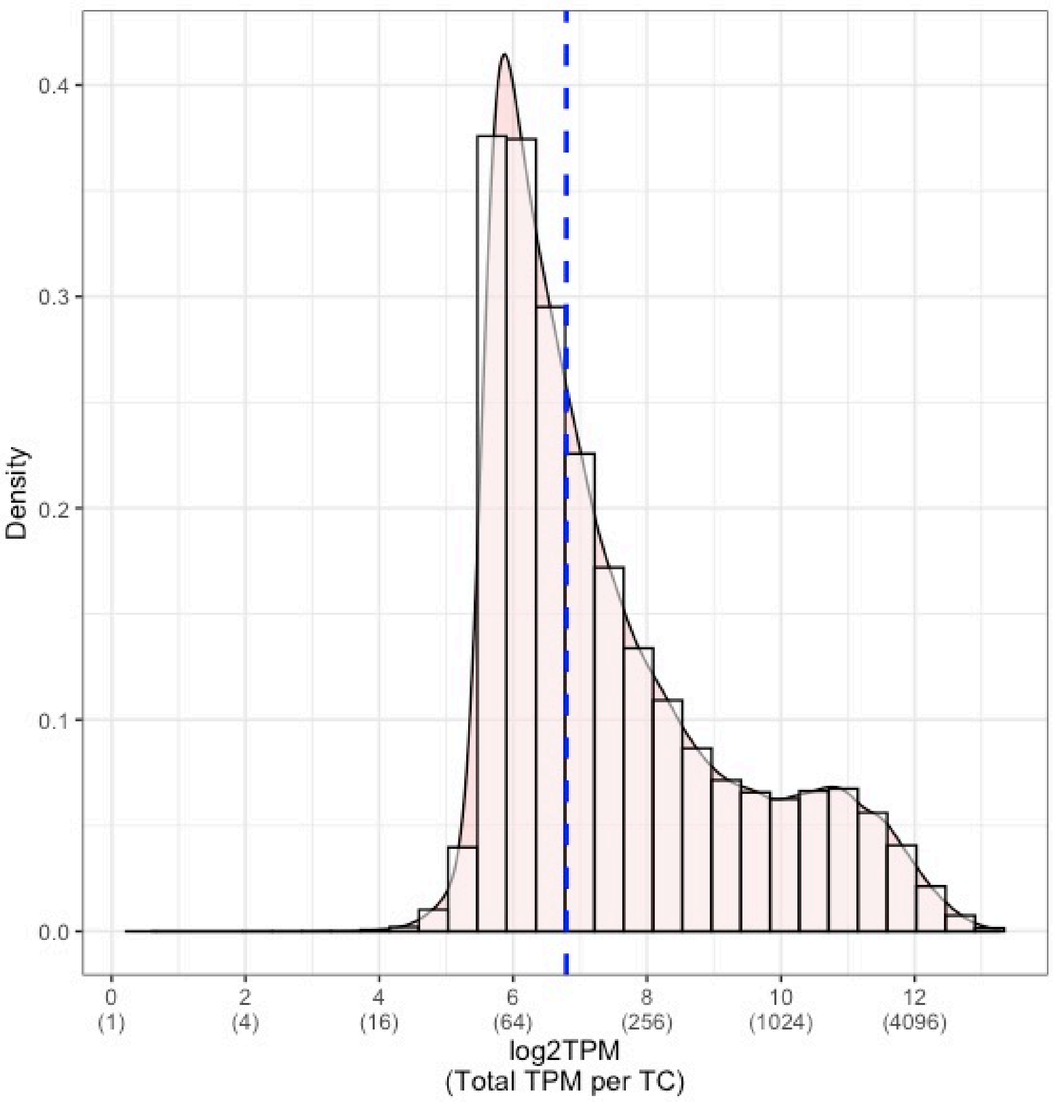

Figure S1: TCs selection. Distribution of TCs based on their expression in log2TPM. The X-axis represents log2TPM and the Y-axis shows the TC density. The blue dotted line corresponds to an average expression cut-off of 3 TPM per sample (37*3=111 TPM in total). TCs on the right side of the dotted line are defined as the robust set.

Tables S1 and S2 | TCs annotation

CAGE TCs are annotated using Ensembl designations to map their locations relative to annotated genes (download date 2020/06).

Table S1: TCs annotation; an excel file contains all CAGE tag clusters (TCs) and their corresponding association to ENSEMBL transcripts, gene symbols, and biotypes (protein-coding, non-coding, etc.).

Features	Comprehensive TCs (n)	Comprehensive TCs (%)	Robust TCs (n)	Robust TCs (%)
IG gene	135	0.17%	92	0.23%
ncRNA	2,060	2.64%	1,178	2.92%
Processed transcript	4,539	5.81%	2,511	6.23%
Protein coding	61,582	78.77%	31,591	78.44%
Pseudogene	1,439	1.84%	798	1.98%
TR gene	168	0.22%	109	0.27%
unknown	8,253	10.56%	3,996	9.92%

Table S2: The genomic location of the CAGE TCs, the comprehensive and the robust set. The distribution of the genomic locations shows ~78% within protein-coding genes, ~5-6% processed transcripts (lncRNA, antisense, non-coding transcript, and so on), ncRNA, and pseudogenes both individually contain ~2%, and ~10% remains unknown. The comprehensive and robust sets are comparable and consistent in terms of their genomic locations.

Figure S2 | Flowchart: A computational workflow

Graphical abstract of the computational workflow.

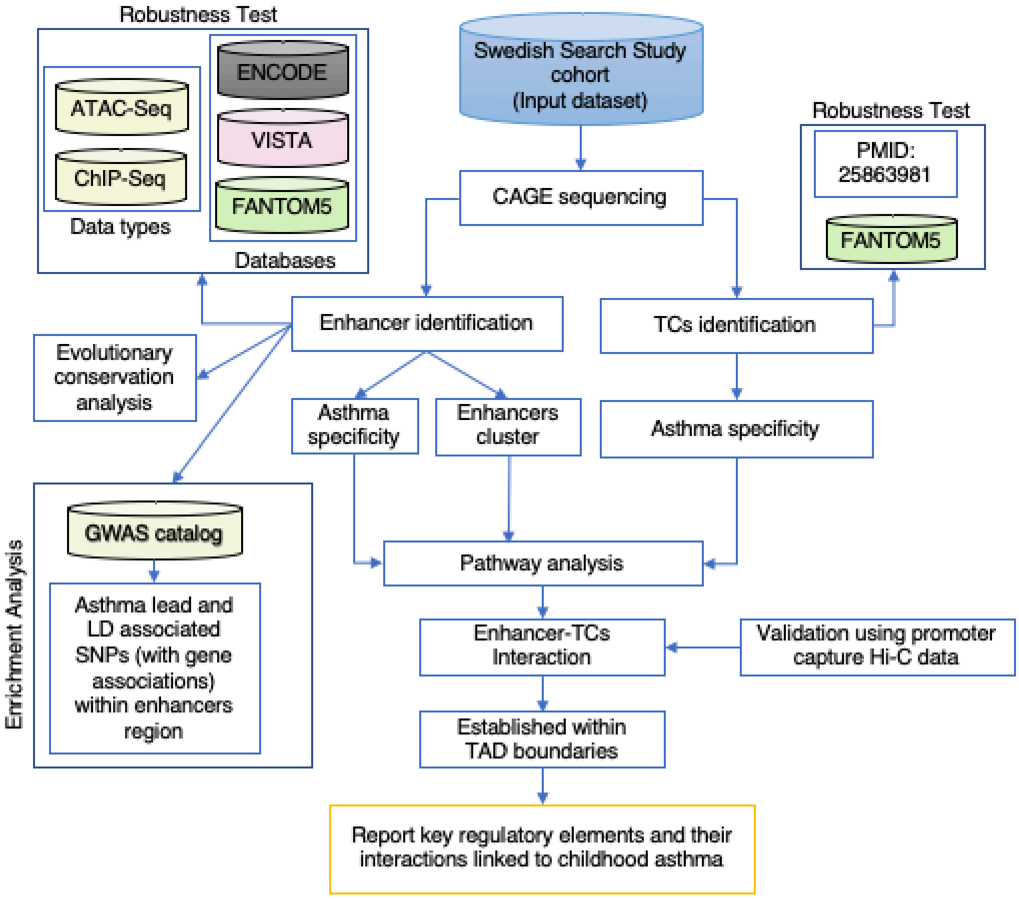

Figure S2: Computational workflow of an integrative analysis to detect asthma-linked enhancers, TCs (Genes), and their interactions from CAGE-seq data.

Figure S3 | Genes, TCs and enhancers in expression signatures

We counted the intersection of our differentially expressed TCs, genes and enhancers in any of the 3 pairwise comparisons.

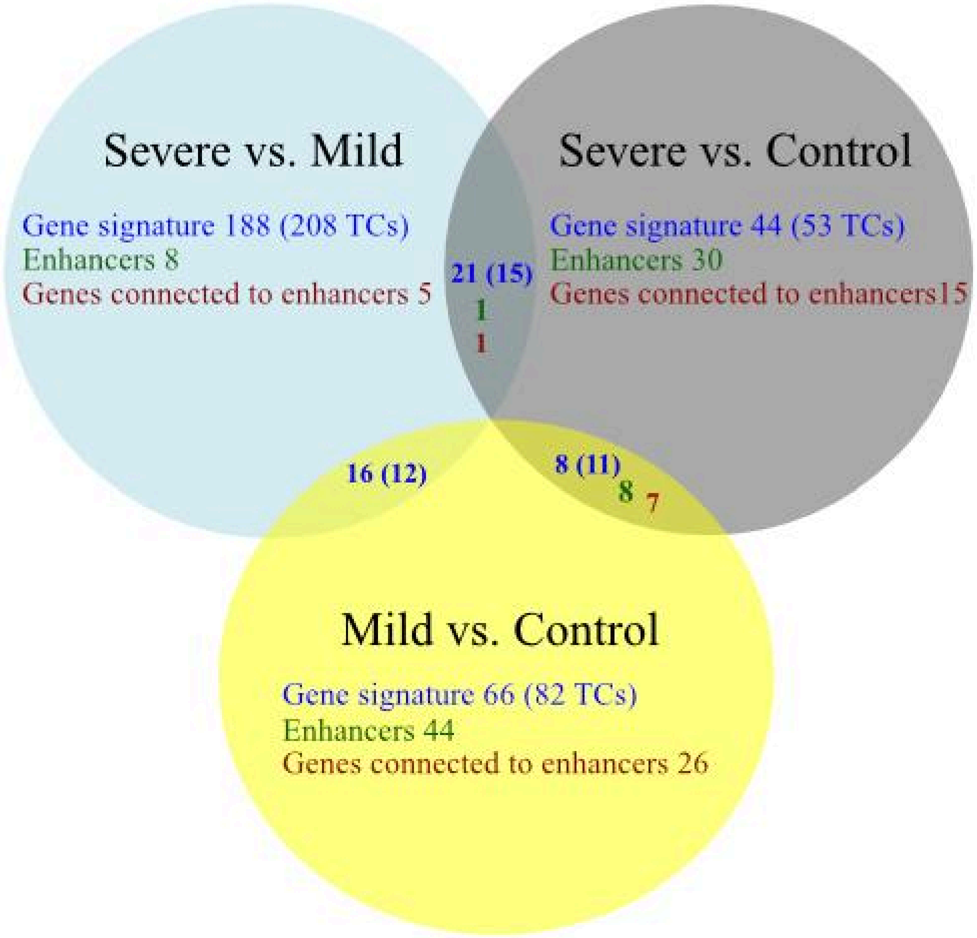

Figure S3: Venn diagram showing the numbers of genes (blue), TCs (blue in brackets) and enhancers (green). Dark red color represents genes connected to enhancers. Note: Two TCs can be associated to the same gene so that the number of genes in the Venn diagram does not add up to the total number of genes considered.

The expression signature consists of 381 TCs corresponding to 321 unique genes.

Group contrast	Genes	Unique genes	Duplicate genes
Severe vs. Control	73	70	3
Mild vs. Control	90	89	1
Severe vs. Mild	225	207	18

Table S3 | Differentially expressed (DE) genes in TCs

CAGE TCs raw count matrices were submitted to EdgeR version 3.28.0 [5] for differential expression (dispersion estimation, and glm-model used) analysis by pairwise comparison among severe, mild, and control asthma. Table S3: DE TCs; an excel file contains only significantly (after FDR correction) up or down-regulated genes or CAGE TCs.

Figure S4 | TCs linked to mild and severe asthma

Volcano plot of all differentially expressed CAGE TCs colored by significance.

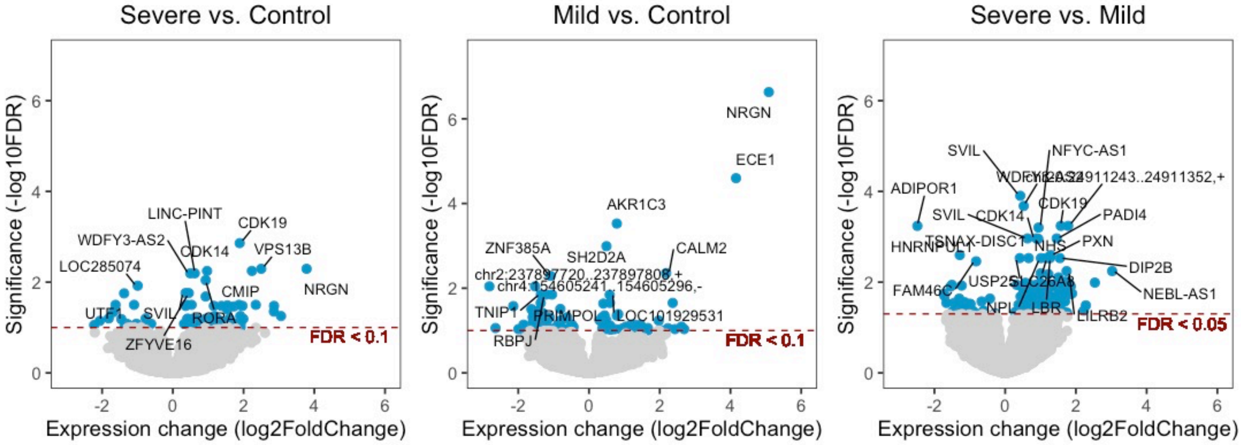

Figure S4: Volcano plot of pairwise differential expression analysis of TCs. The x-axis for expression change in log2 scale and the y-axis for FDR in -log10 scale. TCs (genes) passed a significant level of given FDR highlighted in blue.

Table S4 | WikiPathway analysis of DE TCs

To determine the biological pathways associated with the differentially expressed genes reported in Table S3, we performed the enrichment analysis using Enrichr - an integrative web-based tool [6] through WikiPathways [7]. The enrichment results listed in Table S4, an excel file include all significant (p-value < 0.05) pathways for three pairwise comparisons.

Figure S5 | Test gene expression signatures in an independent dataset

We applied our gene expression signatures to an independent publicly available dataset of gene expression microarray data for CD4+ and CD8+ T cells [8]. These data were from adults with severe and non-severe (mild) asthma. This study showed the widespread change in the activation of CD8+ but not CD4+ T cells from patients with severe asthma. First, we selected all available microarray probes and converted them into gene symbols by using Ensembl biomart. We found 761 probes (panels A and B) and 286 unique probes (panels C and D) for 321 genes (381 TCs) from the expression signature. We performed unsupervised clustering (Pearson correlation, complete linkage clustering) of the expression profiles of the CD4+ and CD8+ T-cell types separately.

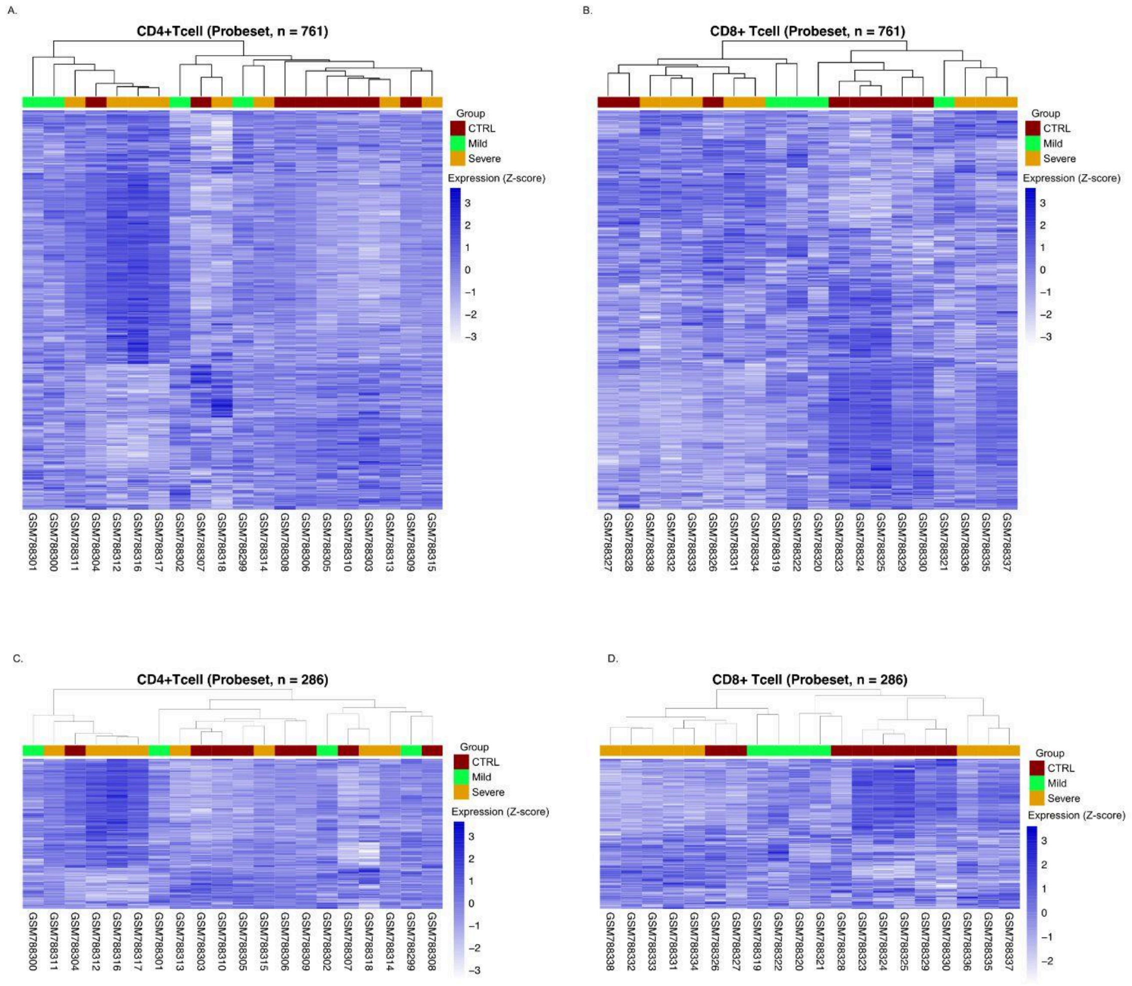

Figure S5: Testing gene expression signatures in an independent dataset. Unsupervised expression clustering was performed for 761 probe sets (several probe sets for the same genes included) from [8] corresponding to the gene expression signature of 321 genes (A and B). Clustering was performed for 286 probes where one probe was selected to represent one gene (C and D).

Figure S6 and Table S5 | Compare TCs to FANTOM5 TCs

We compared our TCs with reported TCs in the FANTOM5 [9]. First, FANTOM5 samples are selected by matching the terms, "CD", "Basophil", "Eosinophil", "Neutrophil", "Macrophage", "Monocyte", "Peripheral", "Whole", and "blood". This resulted in 231 samples. Then took TCs from there for three different settings based on the human blood cell types total CAGE peak expression level, i) >= 4M, ii) >=2M and iii) >=1M tag counts. Figure S4 shows the distribution of tag counts of FANTOM5 samples and Table S4 shows the overlaps between our TCs and the FANTOM5 TCs. Interestingly, after the comparison, we noticed a fewer number of samples with high expression having more TCs.

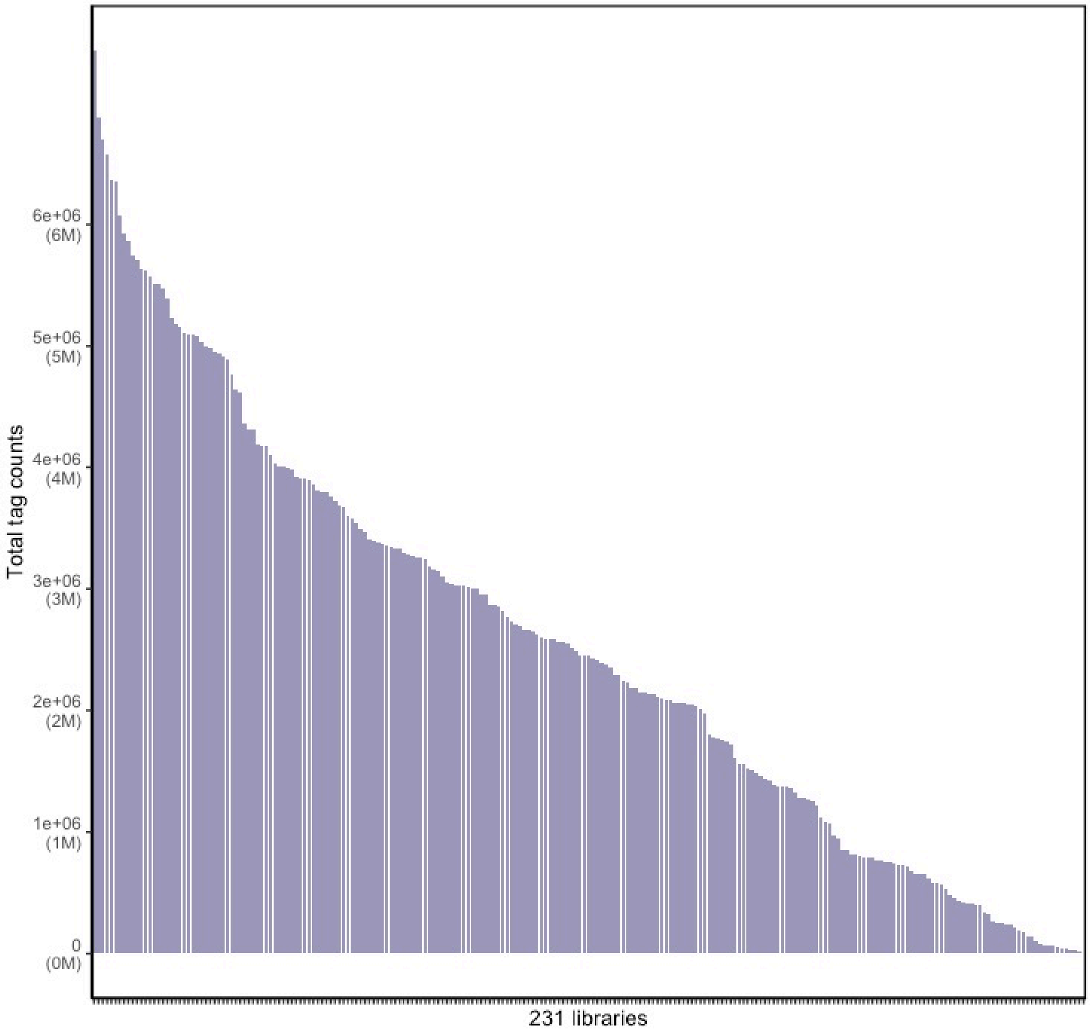

Figure S6: The distribution of tag counts of FANTOM5 samples. After an expression cut-off of 4M, 2M, 1M raw read count per library resulted in 45, 142, and 172 highly expressed libraries, respectively.

	Robust Set	Comprehensive Set
Our TCs	40,273	78,176

FANTOM5	Overlapped	
FANTOM5,<br>total tag counts >=4M,<br>expression cut-off: 3TPM,<br>resulting in:<br>blood samples: 45,<br>TCs: 23,868	18,793 (47%)	20,503 (26%)
FANTOM5,<br>142 blood samples,<br>total tag counts >=2M,<br>TCs = 22,159	18,411 (46%)	19,974 (26%)
FANTOM5,<br>172 blood samples<br>total tag counts >=1M,<br>TCs = 22,111	18,466 (46%)	20,035 (27%)

Table S5: Comparison of identified TCs to the FANTOM5 TCs.

Table S6 | Classification of enhancers

Minimum number of tags per sample	In at least X samples out of 37 samples	Total number of tags at least	Resulting number of enhancers	Comment	Class
2	6	12	4,738	Threshold adopted from Boyd et al. 2018 (PMID: 29695774)	A
10	1	10	1,663	Highly expressed in at least one sample	B
10	2	20	1,248	Highly expressed in at least two samples	C
20	1	20	737	Very highly expressed in at least one sample	D
20	2	40	537	Very highly expressed in at least two samples	E
2	27 (75%)	54	1,306	Present in many samples	F
5	27 (75%)	135	477	Present in many samples	G

Table S6: Classification of enhancers. The detected enhancers were classified in terms of how many samples the enhancers were observed in. The classification letters correspond to Table S6 in the Gitlab repository (Enhancer rawcount table).

Figure S7 | Identification of novel enhancers

All CAGE enhancers were classified according to how many samples they were observed in (Table S6). For further analysis, we followed previously used identification criteria [10] focused on two sets: i) comprehensive enhancers (n= 8,289) with at least 2 tags in 1 sample, and ii) robust enhancers (n= 4,738) with at least 2 tags in 6 samples.

To identify novel enhancers, we compared our CAGE-defined enhancers with the FANTOM5 enhancers [11]. Despite a high level of concordance many enhancers discovered in our comprehensive and robust set were not present in FANTOM5. Taken together, those missing enhancers are termed novel comprehensive and novel robust enhancers.

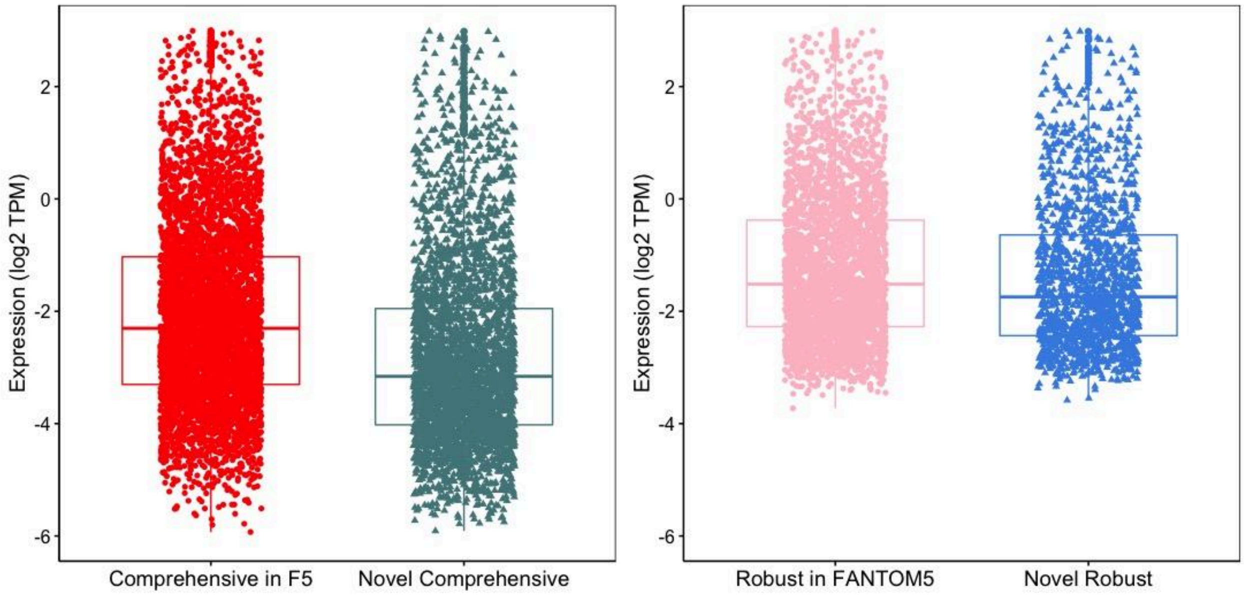

Figure S7: Expression of comprehensive enhancers (n=8,289) and robust enhancers (n=4,738) in all individuals/ samples (n=37). Red color represents comprehensive enhancers (n= 5,012) and pink color represents robust enhancers (n=3,299) found by the FANTOM5 project, cadet blue and bright blue represents novel comprehensive (n=3,277) and novel robust enhancers (n= 1,439) discovered in this work.

Figure S8 | Conservation analysis of enhancer regions

Conservation analysis can be used to annotate the functionality of the genome in all vertebrates [12]. To estimate transcriptional evolutionary conservation with our predicted enhancers, we used UCSC [13] *phyloP100wayAll* track. We randomly sampled 500,000 genomic regions of 4001 bp from the intergenic regions in chromosomes 1 to 22, X, and Y to assess the background level of conservations. Deeptools (computeMatrix command) was used to analyze the correlation among CAGE-defined comprehensive and robust set enhancers, novel comprehensive, novel robust set, and random non-genic region [14].

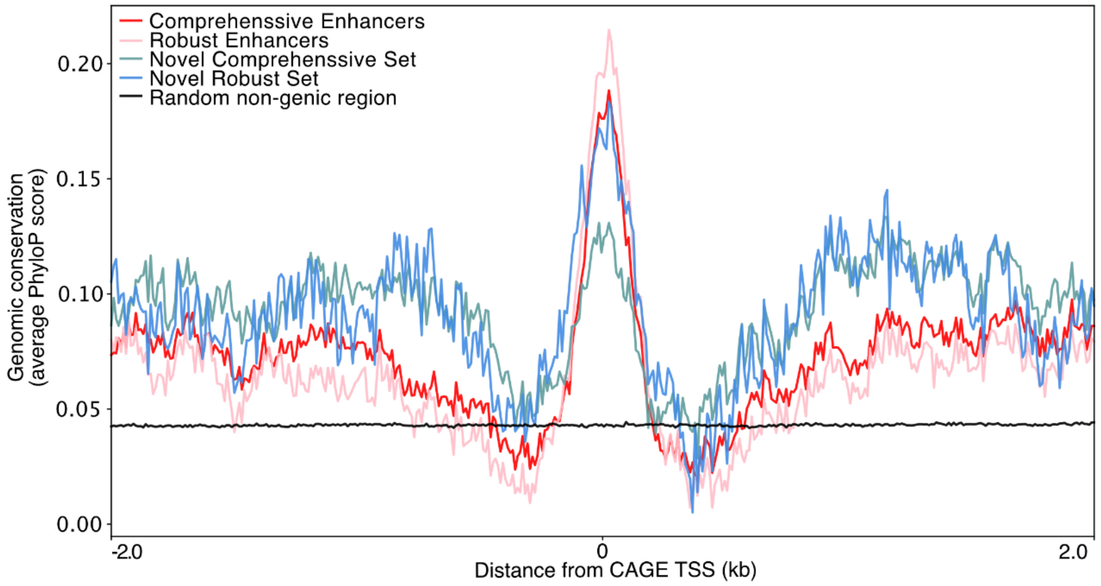

Figure S8: Conservation analysis of enhancer regions. The x-axis shows the distance from the center of regions and the y-axis shows the coverage of PhylopP100 vertebrate conservation score for robust enhancers (n=4,738), comprehensive enhancers (n=8,289), the novel comprehensive enhancers (n= 3,277), novel robust enhancers (n= 1,439) and random non-genic regions (n= 500,000).

Figure S9 | Identification of genomic enhancer cluster

We identified clusters of densely positioned genomic enhancers, which were delineated based on linear nucleotide proximity using a sliding window approach. A window size of 15 kb around the enhancers was selected, and the genomic enhancers cluster was determined using the BEDtools cluster command. Additional 10 kb windows were extended from both the beginning and the end of the genomic enhancers cluster to identify genes that coincide within the genomic enhancers cluster.

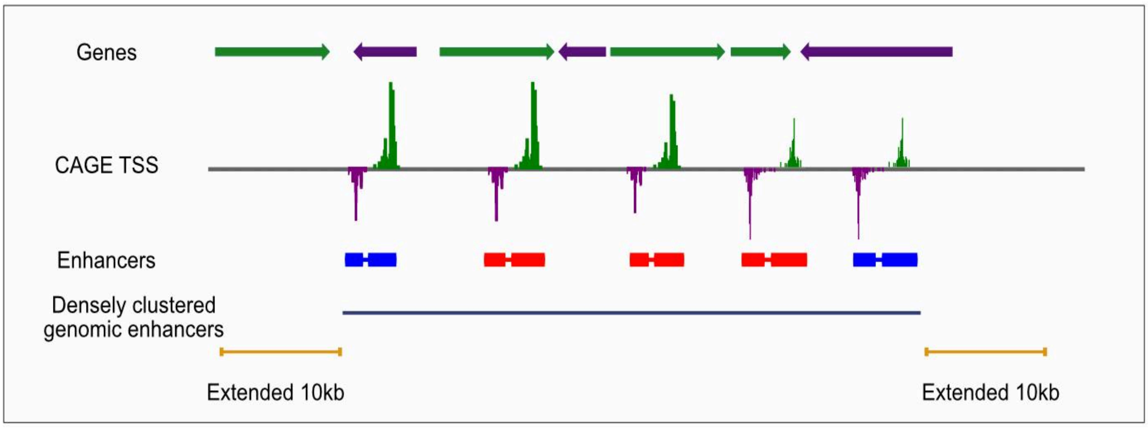

Figure S9: The schematic drawing depicts how genomic enhancers clusters were identified using sliding windows.

Table S7 | Genomic clusters of densely positioned enhancers

The list of genomic enhancer clusters is sorted by cluster size, along with the corresponding genes and GWAS hits SNPs.

Genomic enhancer Cluster Size	Coordinates cluster	Gene Names +/- 10kb	Gene regulated	SNP hits	SNP context
12	chr2:136954140-137023865	*HNRNPKP2*		NO	*NA*
11	chr15:91349622-91427965	*BLM, FURIN, FES*		NO	*NA*
11	chr11:121277812-121353318	*SORL1*		NO	*NA*
11	chr10:126315284-126358304	*FAM53B*		NO	*NA*
10	chr13:99995203-100046916	*UBAC2, FKSG29, MIR623, HMGB3P4*		YES	rs34259893
10	chr2:136860840-136911930	*CXCR4*		NO	*NA*
9	chr19:51616786-51654059	*SIGLEC18P, SIGLEC9, SIGLEC7*		NO	*NA*

9	chr15:67364552-67423883	*SMAD3*		YES	rs1465841, rs117683492
9	chr18:74799887-74836898	*MBP*		NO	*NA*
9	chr10:89840183-89890633	Not found		NO	*NA*
9	chr14:99702959-99746661	*BCL11B*		NO	*NA*
9	chrX:106906230-106964760	*TSC22D3*		NO	*NA*
9	chr9:137248097-137298959	*RXRA, MIR4669*		NO	*NA*
9	chr1:183516531-183588699	*SMG7, NCF2 ,LOC100526839*		NO	*NA*
8	chr1:12200430-12249754	*TNFRSF8, TNFRSF1B*		NO	*NA*
8	chrX:13087180-13120893	Not found		NO	*NA*
8	chr17:38454249-38504759	*CDC6,RARA,RARA-AS1*	SA vs. Ctrl.	NO	*NA*
8	chr19:41804362-41850427	*HNRNPUL1,CCDC97,TGFB1*		NO	*NA*
8	chr21:45553968-45602986	*C21orf33,LOC101928576,LOC101928533*		NO	*NA*
8	chr15:70035784-70071294	Not found		NO	*NA*
8	chr5:86628111-86672890	*RASA1*		NO	*NA*
8	chr13:114884413-114937646	*RASA3*		NO	*NA*
8	chr8:142204028-142260071	*DENND3,SLC45A4*	MA vs.Ctrl	NO	*NA*
8	chr1:150715713-150775936	*CTSS, LOC100132571, CTSK, UBE2D3P3*		NO	*NA*
8	chr4:185730647-185791769	*ACSL1, LOC731424,MIR3945*		NO	*NA*
8	chr1:236015561-236074160	*LYST,MIR1537,RNU5E-2P*		YES	rs18773084
7	chr4:40546103-40598365	*RBM47*		NO	*NA*
7	chr3:46120628-46152699	*Not found*		NO	*NA*
7	chr14:61924208-61967092	*PRKCH*	SA vs. MA	NO	*NA*
7	chr17:72586586-72648288	*CD300LD,C17orf77,LOC101928343,CD300E*		NO	*NA*
7	chr18:74756432-74796045	*MBP*		NO	*NA*
7	chr16:81822545-81885726	*PLCG2*	MA vs.Ctrl	NO	*NA*

7	chr16:84609936-84653569	*COTL1*		NO	*NA*
7	chr10:89812037-89852924	*Not found*		YES	rs1819575
7	chr6:112043095-112095913	*FYN*		NO	*NA*
7	chr11:117863516-117893102	*IL10RA, TMPRSS4-AS1*		NO	*NA*
7	chr11:128365032-128400772	*ETS1,LOC101929517*		NO	*NA*
7	chr3:128936599-128974645	*RPS27P12, COPG1*		NO	*NA*
7	chr8:130973833-131017743	*FAM49B*		NO	*NA*
7	chr8:131191019-131248308	*ASAP1*		YES	rs141721054
7	chr1:167406839-167440327	*CD247,LOC101928512*	MA vs.Ctrl	YES	rs2056625
7	chr5:171590625-171622975	*STK10,EFCAB9*		NO	*NA*
7	chr1:234718683-234758733	*IRF2BP2*		NO	*NA*
7	chr2:238572018-238609578	*LRRFIP1*		NO	*NA*

Table S7: Genomic enhancer cluster coordinates are provided with associated genes and SNPs for clusters containing up to 7 enhancers.

Table S8 | WikiPathway analysis of genomic enhancers cluster

We utilized genomic enhancer clusters (cluster size >= 8) by expanding the window around these clusters by 10 kb and examining the genes in their vicinity. Enrichment analysis of these genes was conducted using Enrichr, an integrative web-based tool (Chen et al., 2013) accessed through WikiPathways (Pico et al., 2008). Table S7 in the supplementary material details the significant pathways (p<0.5) associated with genes from the top 26 clusters.

Term	Overlap	Genes	P.value	Adjusted.P.value	Odds.Ratio
Canonical and Non-Canonical TGF-B signaling WP3874	2/17	*TGFB1;SMAD3*	0.0007	0.025	14.304
Neovascularisation processes WP4331	2/21	*SMAD3; CXCR4*	0.001	0.025	57.788
Vitamin D in inflammatory diseases WP4482	2/22	*SMAD3; RXRA*	0.001	0.025	54.174
Selective expression of chemokine receptors during T-cell polarization WP4494	2/29	*TGFB1; CXCR4*	0.001	0.025	16.355
MAPK Cascade WP422	2/29	*RASA3; MBP*	0.001	0.025	45.613
Extracellular vesicle-mediated signaling in recipient cells WP2870	2/30	*TGFB1;SMAD3*	0.001	0.025	43.33
Cell Cycle WP179	3/120	*SMAD3;TGFB1;CDC6*	0.002	0.031	32.085
Nuclear Receptors WP170	2/38	*RXRA; RARA*	0.002	0.031	32.085
Vitamin A and Carotenoid Metabolism WP716	2/43	*RXRA; RARA*	0.002	0.031	30.938
Interleukin-11 Signaling Pathway WP2332	2/44	*TGFB1;FES*	0.003	0.032	11.302
TGF-beta Receptor Signaling WP560	2/54	*SMAD3;TGFB1*	0.003	0.032	27.065
PPAR signaling pathway WP3942	2/67	*RXRA; ACSL1*	0.004	0.037	24.053
Vitamin D Metabolism WP1531	1/10	*RXRA*	0.004	0.037	22.785
Liver X Receptor Pathway WP2874	1/10	*RXRA*	0.005	0.037	21.115
TGF-beta Signaling Pathway WP366	2/132	*SMAD3;TGFB1*	0.005	0.037	20.611
Mitochondrial LC-Fatty Acid Beta-Oxidation WP368	1/17	*ACSL1*	0.005	0.037	20.611
Platelet-mediated interactions with vascular and circulating cells WP4462	1/17	*TGFB1*	0.006	0.042	18.414
Nuclear Receptors Meta-Pathway WP2882	3/319	*RXRA;TGFB1;TSC22D3*	0.007	0.046	16.966
Farnesoid X Receptor Pathway WP2879	1/19	*RXRA*	0.007	0.046	16.639
Endoderm Differentiation WP2853	2/141	*SMAD3;TGFB1*	0.009	0.055	14.66
NRF2 pathway WP2884	2/146	*RXRA; TGFB1*	0.011	0.063	13.302

Table S8:Pathway analysis (p<0.5) was conducted for 48 genes in the vicinity of the top 26 genomic enhancer clusters.

Table S9 | Genomic enhancers cluster on chromosome 2

Genomic enhancers cluster on chromosome 2 (chr2:136430415-137327374)<br>Genes: *R3HDM1,UBXN4,LCT,LOC100507600,MCM6,LOC391448,DARS,DARS-AS1,CXCR4,HNRNPKP2, UBBP1 and LOC100421125*					
**Term**	**Overlap**	**Genes**	**P.value**	**Adjusted.P.value**	**Odds.Ratio**
Alanine and aspartate metabolism WP106	1/12	*DARS*	0.007	0.063	165.099
Amplification and Expansion of Oncogenic Pathways as Metastatic Traits WP3678	1/17	*CXCR4*	0.01	0.063	113.477
Neovascularisation processes WP4331	1/21	*CXCR4*	0.013	0.063	90.764
Selective expression of chemokine receptors during T-cell polarization WP4494	1/29	*CXCR4*	0.017	0.065	64.805
DNA Replication WP466	1/42	*MCM6*	0.025	0.07	44.228
Cardiac Progenitor Differentiation WP2406	1/53	*CXCR4*	0.031	0.07	34.853
Hematopoietic Stem Cell Differentiation WP2849	1/55	*CXCR4*	0.033	0.07	33.559
G1 to S cell cycle control WP45	1/64	*MCM6*	0.038	0.07	28.752
Peptide GPCRs WP24	1/74	*CXCR4*	0.044	0.07	24.801
Viral Acute Myocarditis WP4298	1/84	*CXCR4*	0.049	0.07	21.802

Table S9: Pathway analysis for genomic enhancers cluster on chromosomes 2.

Table S10 | GWAS asthma lead and LD associated SNPs (with gene associations) within enhancers region

Table S10, an excel file, has listed GWAS asthma lead SNPs and LD associated SNPs that were located within enhancer regions. To establish SNPs within the enhancer regions we considered the disease traits in Table 2 as well as the following by choosing a genomic region of 500 bp upstream of the 5’ end of the enhancers and 500 bp downstream of the 3’ end of the enhancers. We explored 12 different traits in our search for SNPs associated with asthma. Unfortunately, enhancer regions did not align with the following traits:

- Response to corticosteroid
- Response to Montelukast
- Response to zileuton
- Respiratory symptom change measurement
- FVC change measurement
- FEV change measurement

Interestingly, we found SNPs within enhancer regions only for traits from Table 2.

Figure S10 | Genes are regulated by enhancers

(A) *SBNO2*
SBNO2, an asthma-relevant gene, is linked to an enhancer.

(A) The upper panel displays the Zenbu genome browser featuring UCSC gene models. CAGE TSS expression is depicted for both strands: green indicates the forward strand and purple the reverse strand. All identified TCs and enhancers are visualized, with red representing novel enhancers, blue for robust enhancers, and black for comprehensive enhancers. Pink interaction lines connect significantly correlated TCs and enhancers. The zoom-in box illustrates the expression of this enhancer, which potentially regulates *SBNO2*. This enhancer is not only novel but also part of a genomic cluster of densely positioned enhancers. The blue line delineates the range of this genomic enhancer cluster, which contains GWAS SNPs, including one childhood-onset asthma SNP (light blue colored). Asthma-related GWAS lead SNPs and LD-associated SNPs are shown in brown.

(B, C) The middle panel displays the expression in various cell types from FANTOM5, with *SBNO2* on the left side and the enhancer on the right.

(D, E) The lower panel presents the expression analysis from our CAGE data. The lower-left plot demonstrates the correlation between TC (*SBNO2*) and the enhancer. The lower-middle and right plots depict the expression in TPM for control, MA, and SA in a box plot for TC (*SBNO2*) and the enhancer, respectively.

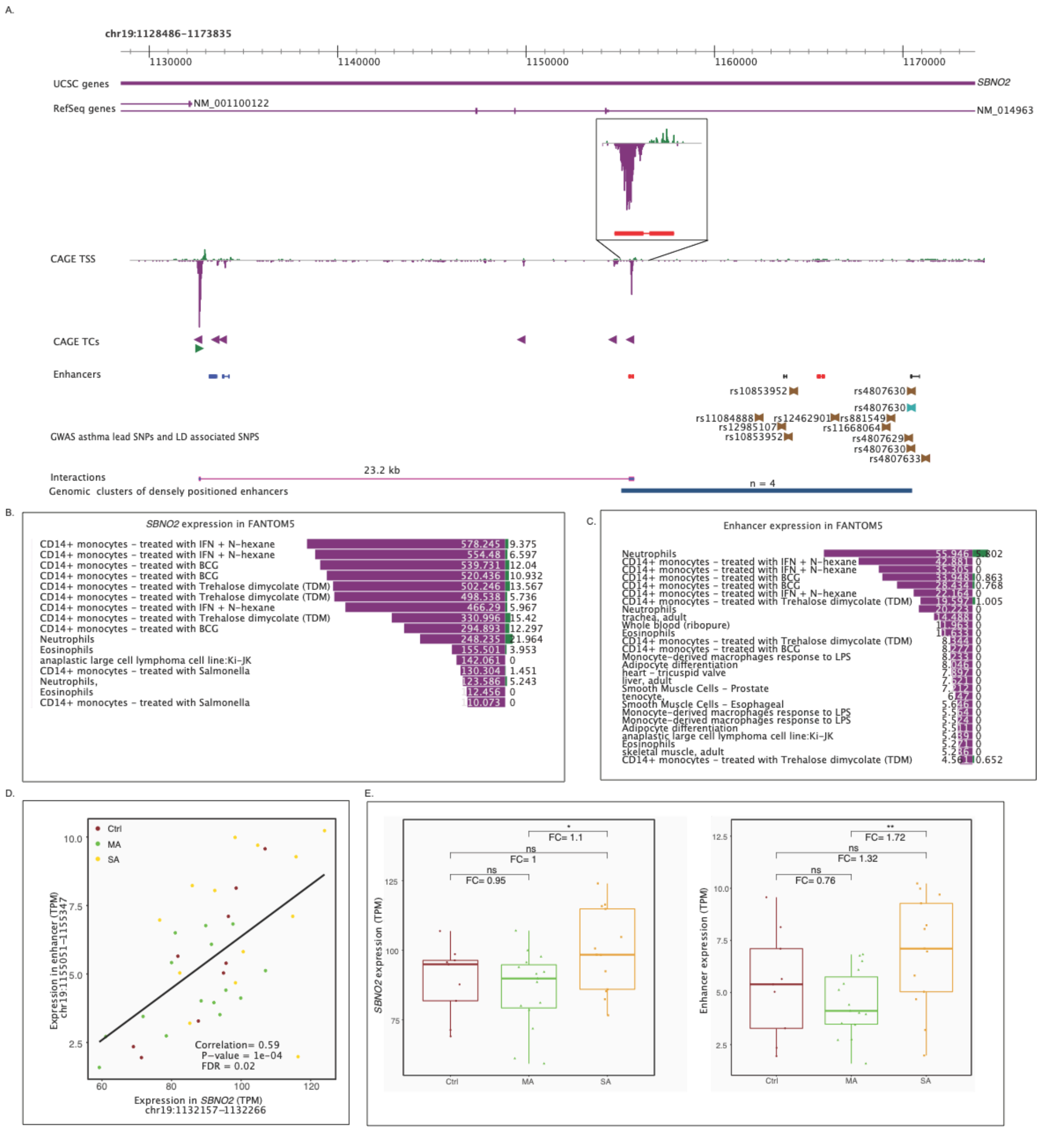

(B) *SLC19A1*

SLC19A1 is an example of a lung function (FEV1/FVC)-related gene linked with (possibly regulated by) our CAGE TCs and enhancers.

(A) The upper panel depicts the Zenbu genome browser with UCSC gene models, showing CAGE TSS expression for both strands: green for the forward strand and purple for the reverse strand. All identified TCs and enhancers are visualized. Significantly correlated TCs and enhancers are connected by a pink interaction line. The zoom-in box displays the expression of this enhancer, which might regulate *SLC19A1*. Asthma-related GWAS lead SNPs and LD-associated SNPs are shown in brown. This enhancer is a robust enhancer close to a GWAS SNP linked with lung function (FEV1/FVC).

(B, C) The middle panel shows the expression in different cell types from FANTOM5, with *SLC19A1* on the left side and the enhancer on the right.

(D, E) The lower panel shows the expression analysis from our CAGE data. The lower-left plot demonstrates the correlation between the TC (*SLC19A1*) and the enhancer. The

lower-middle and right plots display the expression in TPM for control, MA, and SA in a box plot for TC (*SLC19A1*) and the enhancer, respectively.

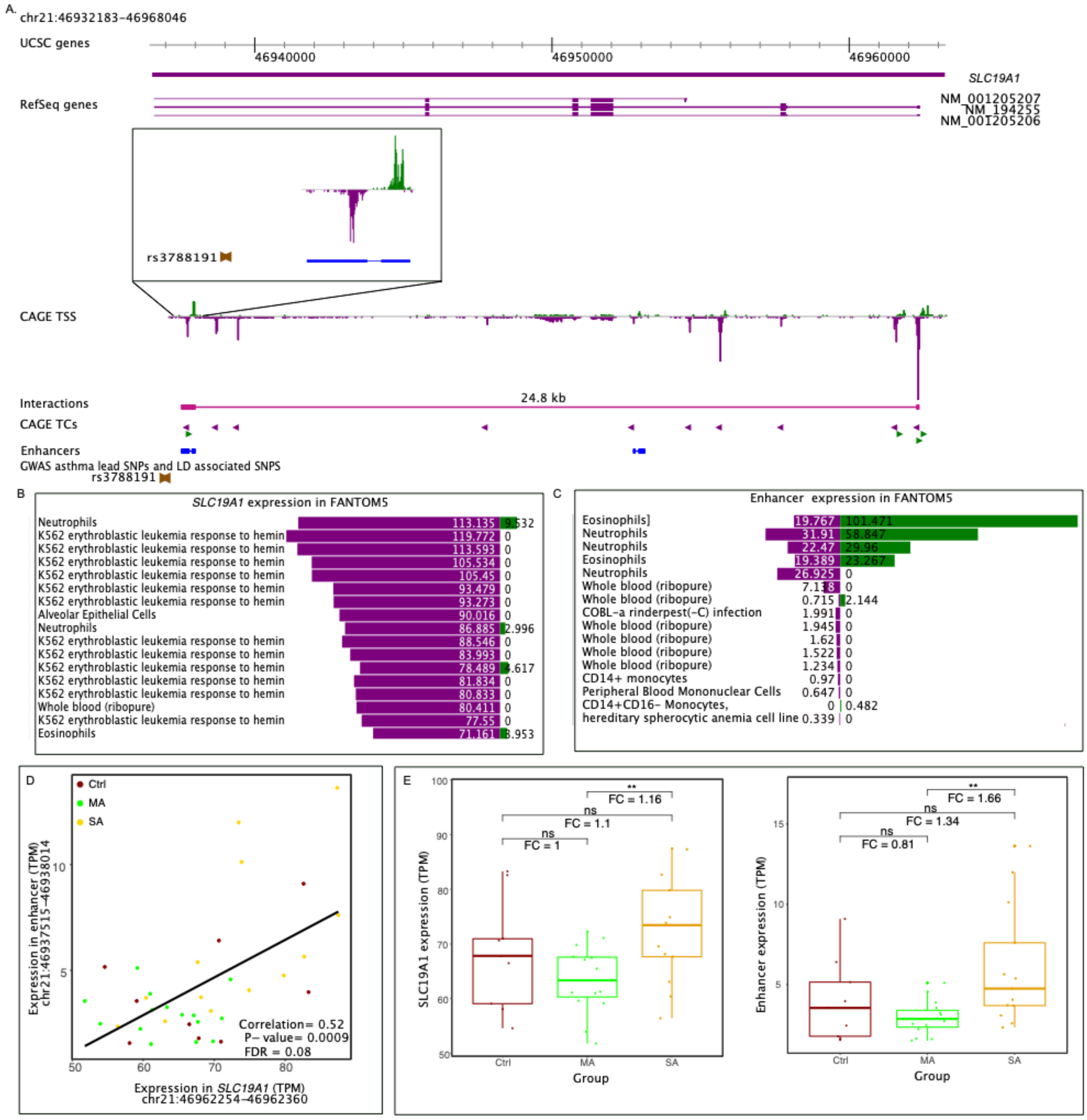

(C) *CXCL1*

CXCL1 is regulated by two enhancers in neutrophils.

(A) The upper panel depicts the Zenbu genome browser for gene models with UCSC genes and RefSeq genes. It shows CAGE TSS expression for both strands: green for the forward strand and purple for the reverse strand. All identified TCs and enhancers are visualized. Significantly correlated TCs and enhancers are connected by a pink interaction line. The zoom-in box displays the expression of this enhancer, which might regulate *CXCL1*. This enhancer is a robust enhancer.

(B, C) The middle panel shows the expression in different cell types from FANTOM5, with *CXCL1* on the left side and the enhancer on the right.

(D) The lower panel shows the expression analysis from our CAGE data. The lower-left plot demonstrates the correlation between the TC (*CXCL1*) and the enhancer. The lower-middle and right plots display the expression in TPM for control, MA, and SA in a box plot for TC (*CXCL1*) and the enhancer, respectively.

CXCL1 gene was described in the context of asthma [15] [16]. *CXCL1* is expressed broadly in many cell types and tissues including smooth muscle and lymphatic endothelial cells (Figure S14). *CXCL1* expression was elevated in children with severe asthma compared to mild asthma. We identified two robust enhancers, 3.6 kbp and 5.4 kbp downstream of *CXCL1*, both of which displaying distinct bi-model and bi-directional expression patterns characteristic for enhancers. Both enhancers were expressed specifically in neutrophils and CD14+ monocytes and expression in children with severe asthma was elevated. Moreover, the expression of these two enhancers correlated with the expression of *CXCL1* indicating that the gene might be regulated by both enhancers in neutrophils and CD14+ monocytes.

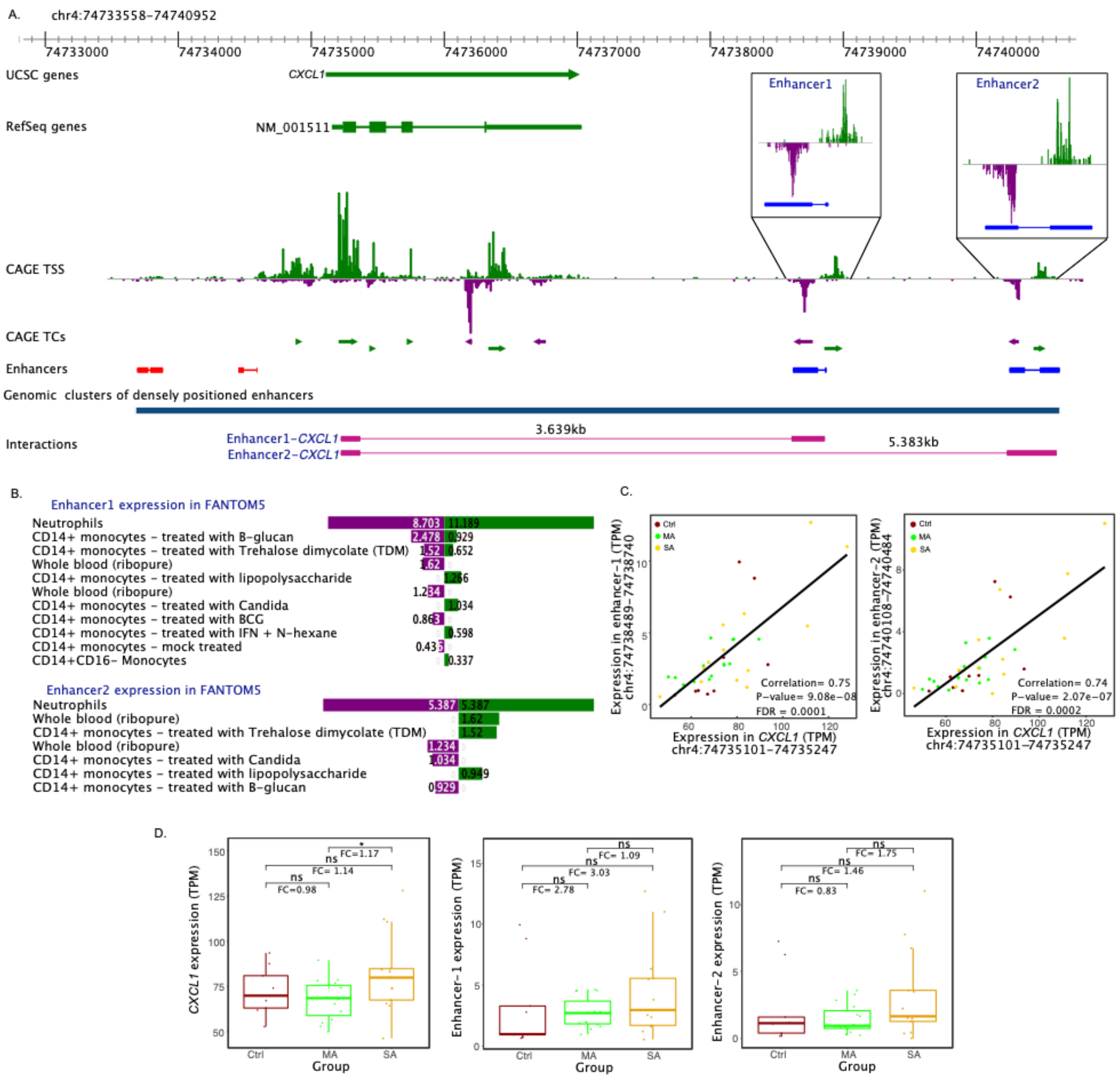

CXCL1 gene is possibly regulated by two enhancers. Two enhancers were identified downstream of *CXCL1* (A) which were expressed in neutrophils and CD14+ monocytes (B). Expression of both enhancers correlated with *CXCL1* expression (C). *CXCL1* as well as both enhancers are elevated in expression in children with severe asthma (D).

(D) *VAV3*
VAV3, an asthma-relevant gene, is linked to (regulated by) an enhancer.

(A) The upper panel depicts the Zenbu genome browser with UCSC gene models. CAGE TSS expression is shown for both strands: green for the forward strand and purple for the reverse strand. All identified TCs and enhancers are visualized (red for novel, blue for robust, and black for comprehensive enhancers). Significantly correlated TCs and enhancers are connected by a light blue interaction line. The zoom-in box displays the expression of this enhancer, which might regulate *VAV3*. This enhancer is a robust enhancer. Asthma-related GWAS lead SNPs and LD-associated SNPs are shown in brown. Within a 10 kb window from the enhancer, there are three GWAS SNPs found. These SNPs are related to the response to bronchodilators, with distances from the enhancer of 8.4 kb, 5.2 kb, and 6.6 kb (brown colored). Promoters capture HiC data in different cell lines is also shown.

(B, C) The middle panel shows the expression in different cell types from FANTOM5, with *VAV3* on the left side and the enhancer on the right.

(D) The lower panel shows the expression analysis from our CAGE data. The lower-left plot demonstrates the correlation between TC (*VAV3*) and the enhancer. The lower-middle and right plots display the expression in TPM for control, MA, and SA in a box plot for TC (*VAV3*) and the enhancer, respectively.

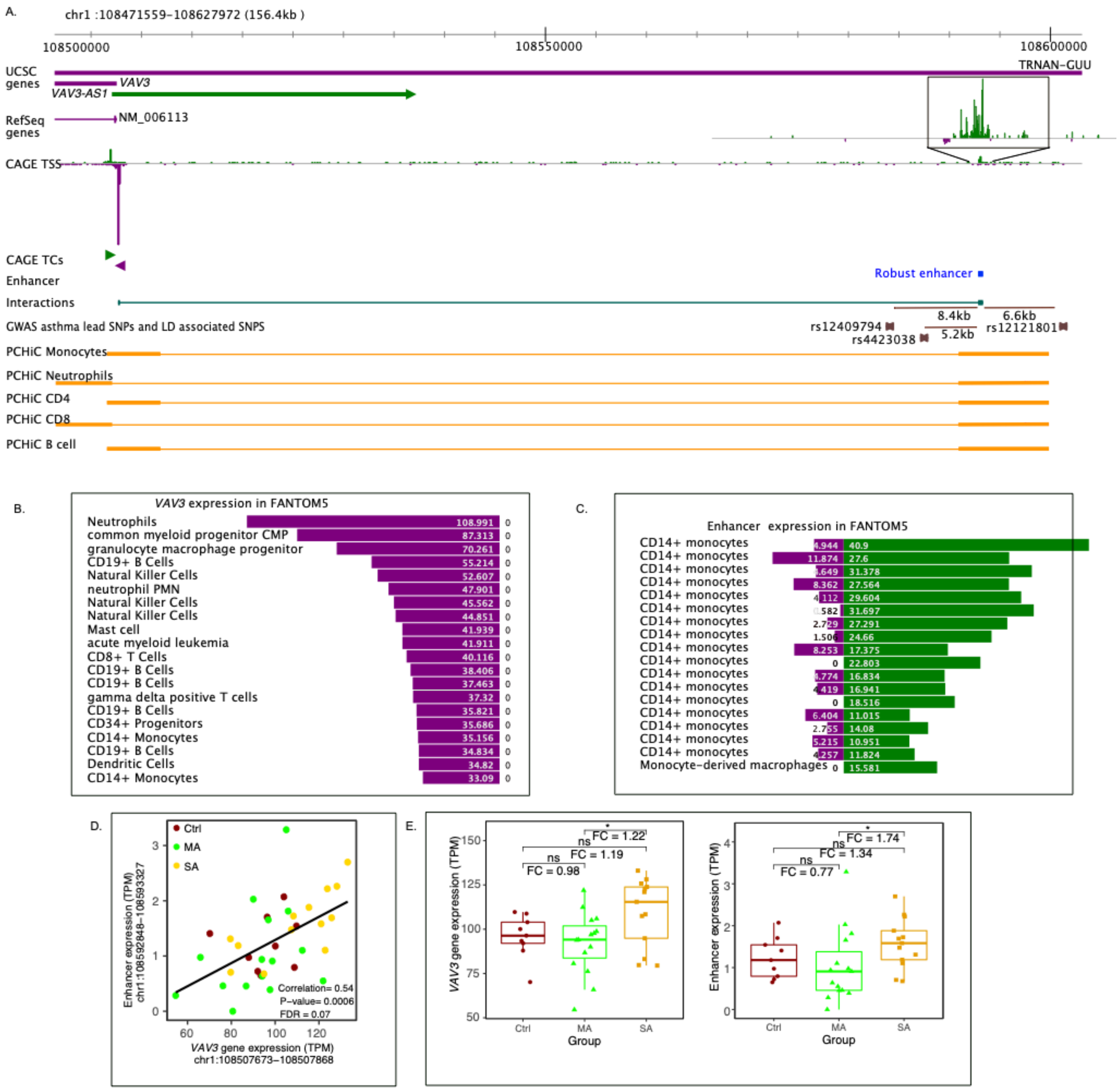
A.
chr1 :108471559-108627972 (156.4kb)
108500000
108550000
108600000
TRNAN-GUU
UCSC genes
VAV3
VAV3-AS1
RefSeq genes
NM_006113
CAGE TSS
CAGE TCs
Enhancer
Robust enhancer
Interactions
8.4kb
6.6kb
5.2kb
GWAS asthma lead SNPs and LD associated SNPS
rs12409794
rs4423038
rs12121801
PCHiC Monocytes
PCHiC Neutrophils
PCHiC CD4
PCHiC CD8
PCHiC B cell
B.
VAV3 expression in FANTOM5
Neutrophils 108.991 0
common myeloid progenitor CMP 87.313 0
granulocyte macrophage progenitor 70.261 0
CD19+ B Cells 55.214 0
Natural Killer Cells 52.607 0
neutrophil PMN 47.901 0
Natural Killer Cells 45.562 0
Natural Killer Cells 44.851 0
Mast cell 41.939 0
acute myeloid leukemia 41.911 0
CD8+ T Cells 40.116 0
CD19+ B Cells 38.406 0
CD19+ B Cells 37.463 0
gamma delta positive T cells 37.32 0
CD19+ B Cells 35.821 0
CD34+ Progenitors 35.686 0
CD14+ Monocytes 35.156 0
CD19+ B Cells 34.834 0
Dendritic Cells 34.82 0
CD14+ Monocytes 33.09 0
C.
Enhancer expression in FANTOM5
CD14+ monocytes 40.9
CD14+ monocytes 11.874 27.6
CD14+ monocytes 31.378
CD14+ monocytes 8.362 27.564
CD14+ monocytes 29.604
CD14+ monocytes 31.697
CD14+ monocytes 27.291
CD14+ monocytes 24.66
CD14+ monocytes 8.253 17.375
CD14+ monocytes 0 22.803
CD14+ monocytes 16.834
CD14+ monocytes 16.941
CD14+ monocytes 0 18.516
CD14+ monocytes 6.404 11.015
CD14+ monocytes 14.08
CD14+ monocytes 5.215 10.951
CD14+ monocytes 11.824
Monocyte-derived macrophages 0 15.581
D.
Ctrl
MA
SA
Enhancer expression (TPM) chr1:108592848-108593327
Correlation= 0.54
P-value= 0.0006
FDR = 0.07
VAV3 gene expression (TPM) chr1:108507673-108507868
E.
VAV3 gene expression (TPM)
FC = 1.22
FC = 1.19
FC = 0.98
ns
Ctrl
MA
SA
Group
Enhancer expression (TPM)
FC = 1.74
FC = 1.34
FC = 0.77
ns

(E) *PTEN*

PTEN, an airway hyperresponsiveness-relevant gene, is linked to (regulated by) an enhancer.

(A) The upper panel shows the Zenbu genome browser with UCSC gene models. CAGE TSS expression is displayed for both strands: green for the forward strand and purple for the reverse strand. All identified TCs and enhancers are visible (red for novel, blue for robust, and black for comprehensive enhancers). Significantly correlated TCs and enhancers are linked by a light blue interaction line. The zoom-in box illustrates the expression of this enhancer, which may regulate *PTEN*. Several enhancers are robust and novel. The blue line indicates the range of this cluster of densely positioned enhancers, which contains GWAS asthma lead SNPs and LD-associated SNPs related to FEV/FVC (brown colored). Promoter capture HiC data from CD4 cell lines is also included.

(B,C) The middle panel shows the expression in different cell types from FANTOM5, with *PTEN* on the left side and the enhancer on the right.

(D) The lower panel shows the expression analysis from our CAGE data. The lower-left plot demonstrates the correlation between TC (*PTEN*) and the enhancer.

(E) The lower-middle and right plots show the expression in TPM for control, MA, and SA in a box plot for TC (*PTEN*) and the enhancer, respectively.

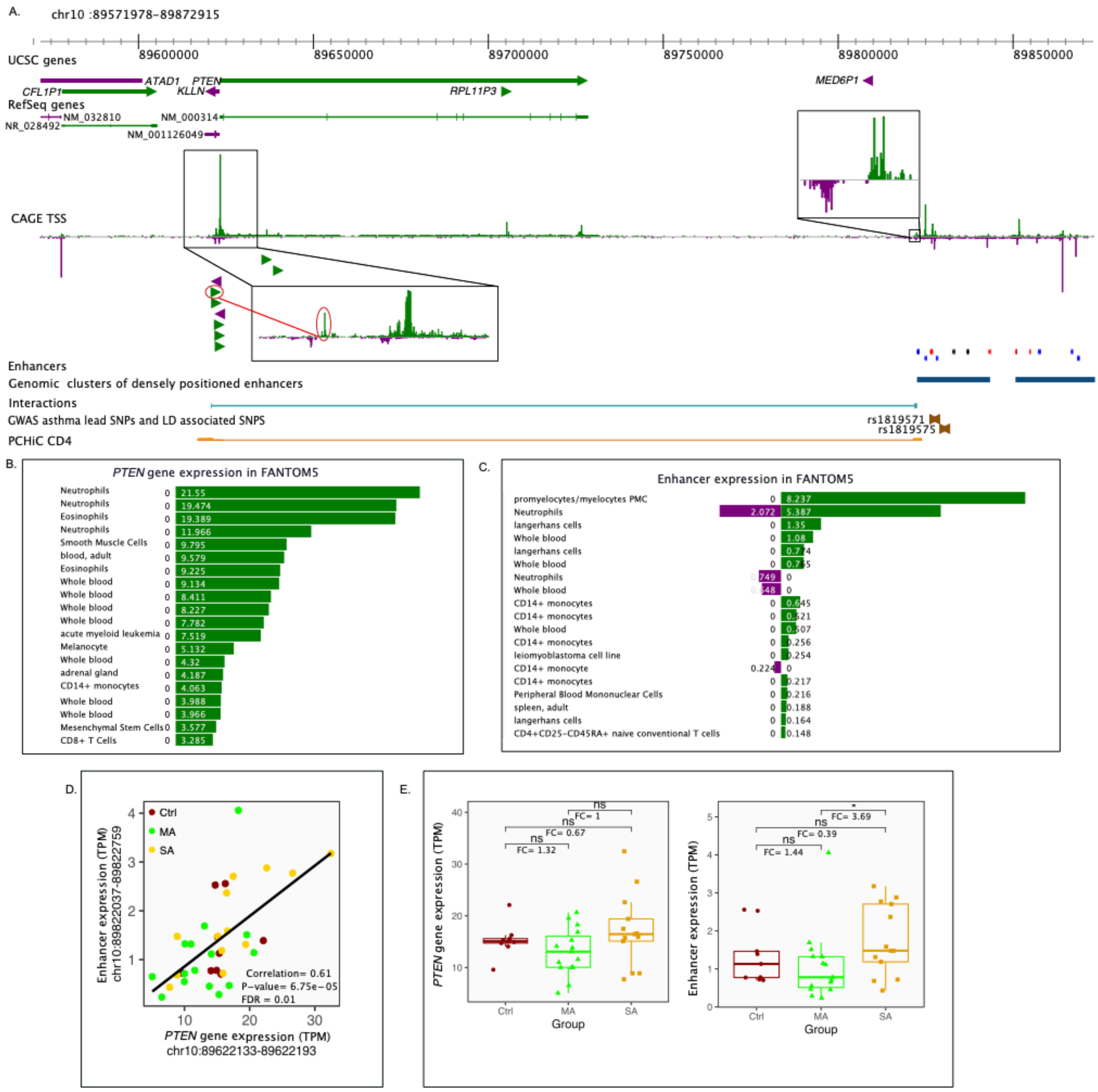
A.
chr10 :89571978-89872915
UCSC genes
RefSeq genes
CAGE TSS
Enhancers
Genomic clusters of densely positioned enhancers
Interactions
GWAS asthma lead SNPs and LD associated SNPS
PCHiC CD4
rs1819571
rs1819575
B.
PTEN gene expression in FANTOM5
C.
Enhancer expression in FANTOM5
D.
Correlation= 0.61
P-value= 6.75e-05
FDR = 0.01
PTEN gene expression (TPM)
chr10:89622133-89622193
Enhancer expression (TPM)
chr10:89822037-89822759
E.
PTEN gene expression (TPM)
Enhancer expression (TPM)
Group

Figure S11 | Enhancers linked to severe and mild asthma

Differentially expressed enhancers are identified in three contrasts shown in volcano plots.

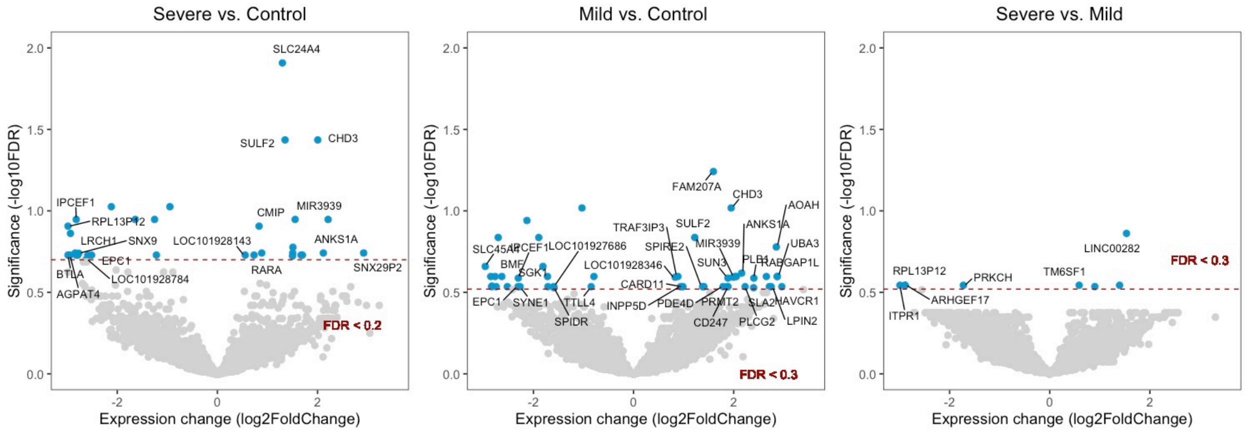

Figure S11: Differential expression analysis of enhancers using glm. The volcano plots show differentially expressed enhancers. The x-axis depicts the expression change in the log2 scale, the y-axis depicts the significance (FDR) of the fold changes in the -log10 scale. Enhancers are named by gene names of the genes closest to the respective enhancer. Enhancers (closest genes) passed given significant levels highlighted in blue. Severe vs. control: 39 enhancers; mild vs. control: 52 enhancers Severe vs. mild: 9 enhancers.

Table S11 | Differentially expressed (DE) enhancers

CAGE-defined enhancer raw count matrices were used as input for EdgeR to estimate the differential expression by pairwise comparison among severe, mild, and controlled asthma. Table S11, an excel file provides only significantly up or down-regulated enhancers.

Table S12 | WikiPathway analysis of DE enhancers

The enrichment analysis used Enrichr - an integrative web-based tool [6] through WikiPathways [7] for the differentially expressed enhancer-related gene list. Table S12, an excel file stored all significant (p-values < 0.05) pathways for three pairwise comparisons.

Figure S12 | Hierarchical clustering in severe and mild asthma

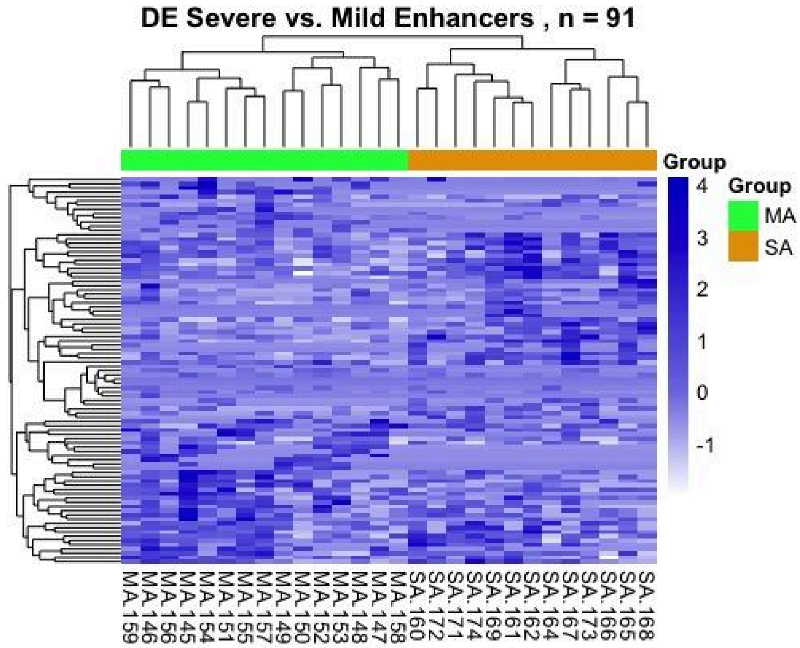

Figure S12: Enhancer signature from a direct contrast between SA and MA. An enhancer signature of 91 enhancers distinguished between individuals with SA and MA when the comparison was performed between these two groups directly.

Figure S13 | Identify enhancer-TC interactions

Flow chart illustrating how we identified possible interactions between enhancers and TCs .

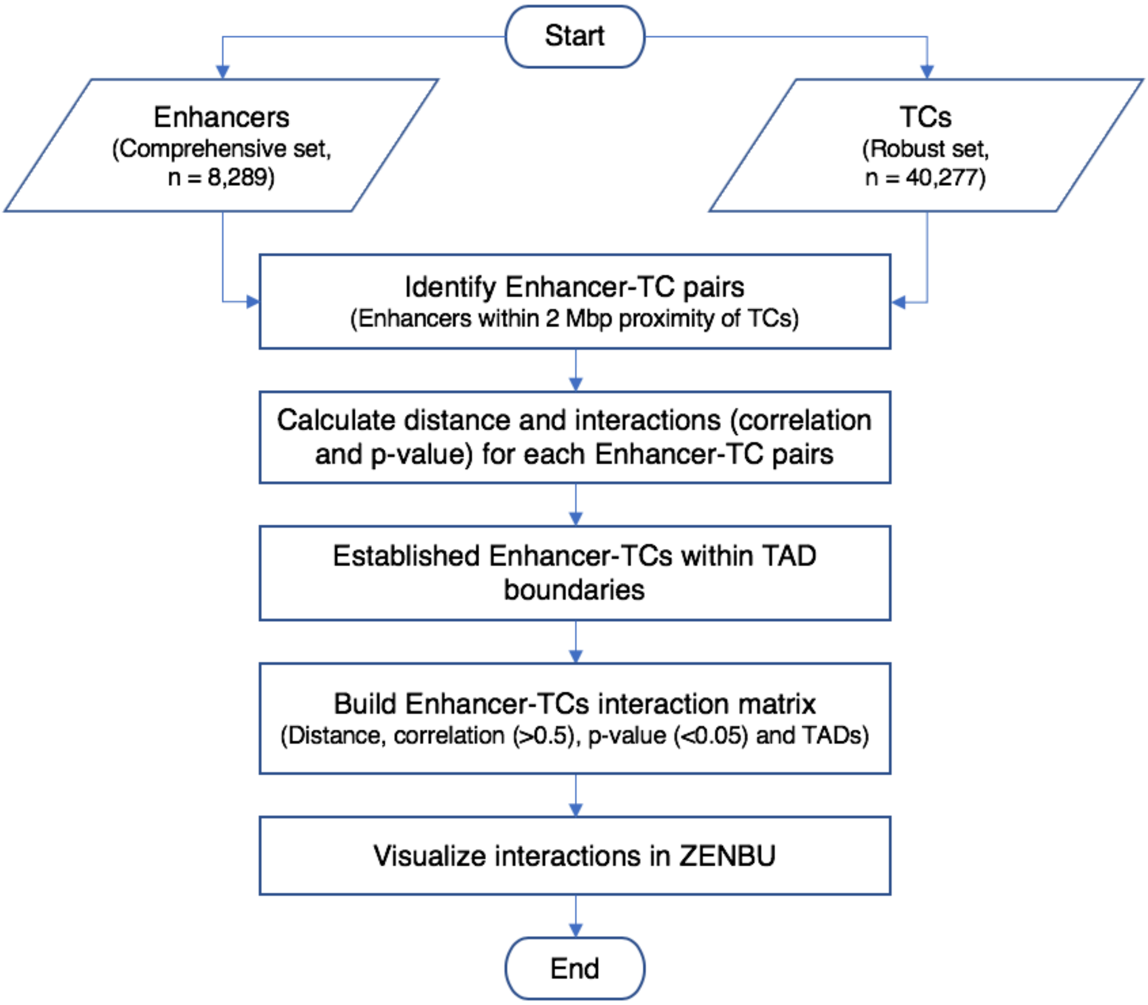

Figure S13 (a): Work-flow to identify possible regulatory interactions between enhancer and tag-clusters (TCs).

We visualized the relationship between R and FDR for our enhancer-gene interactions (Figure S13B). R>=0.5 corresponded to FDR <= 0.125, and R>=0.55 corresponded to FDR<0.05.

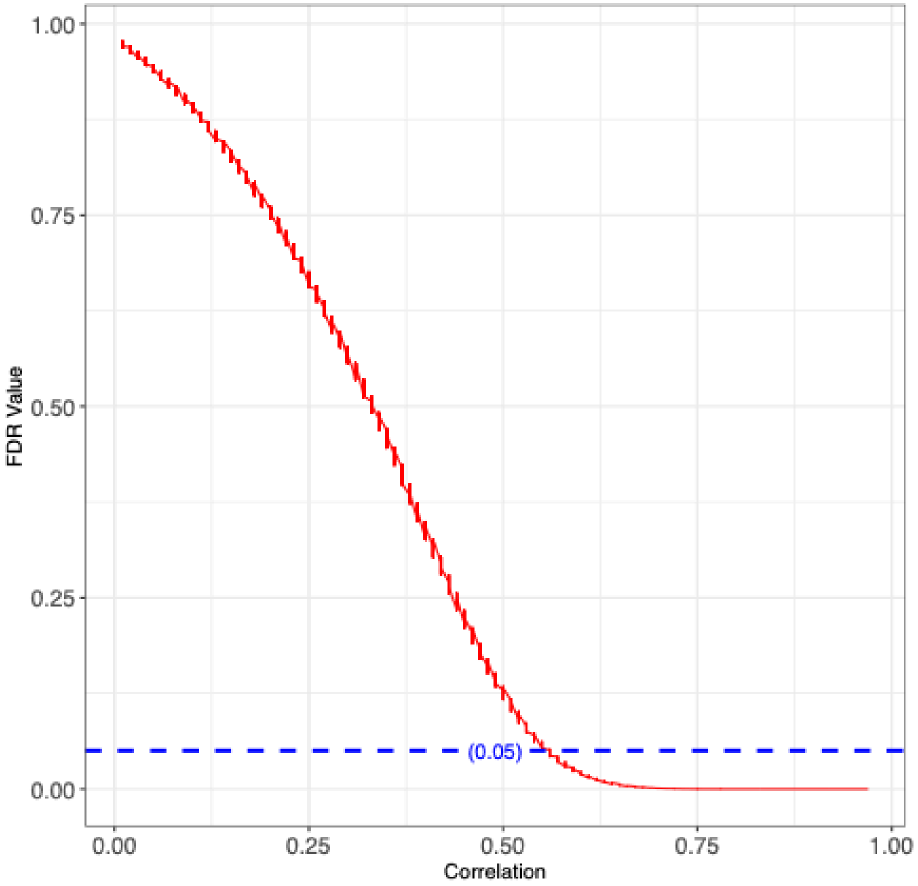

Figure S13(b): Relationship of FDR values and correlation coefficients for enhancer-gene interaction candidates. Enhancer-gene interactions with R>=0.50 are provided in Table S13.

Table S13 | Genes are regulated by enhancers

After identifying possible enhancer-TCs interactions (as explained in the above Figure S10), we established the TAD boundaries by using the following TAD datasets:

- Dataset 1: Hi-C loops [17], GEO accession number GSE115896.
  GSM3967114_T_cell_1_TADs_hicratio-500kb-activity.bed.gz
  GSM3967115_T_cell_2_TADs_hicratio-500kb-activity.bed.gz
  GSM3967116_T_cell_3_TADs_hicratio-500kb-activity.bed.gz

- Dataset 2: Hi-C loops by the Yue lab [18](http://yuelab.org/).
  hg19.TADs only lung donor (Lung_Donor-LG1-raw_TADs.txt)

- Dataset 3: Hi-C data loops [19](https://osf.io/u8tzp/).
  TADs_Mon_mean_merged.bed
  TADs_nB_mean_merged.bed
  TADs_nCD4_mean_merged.bed
  TADs_nCD8_mean_merged.bed
  TADs_Neu_mean_merged.bed

Note: Comprehensive TCs and robust enhancers were used to define TAD boundaries (BEDtools intersect command).

Table S13, an excel file lists 5056 possible enhancer-TCs interactions within TAD boundaries.

Table S14 | Enhancers are intersecting with genes

Table S14 shows our interactions confirmed by publicly available promoter capture Hi-C from several populations of human leukocytes to provide some experimental support for enhancer-gene interactions. We extended the section “Enhancers possibly regulate expression of asthma genes” and corresponding Table 3 by reporting the support of identified enhancer-gene interactions with promoter capture Hi-C data [20].

Overlap	Monocytes	Neutrophils	CD4	CD8	B-cell	Any cell type
Robust TCs (3,664) with bait ends	1,140 (31.1%)	1,115 (30.4%)	1,182 (32.2%)	1,174 (32.0%)	1,084 (29.5%)	1,404 (38.3%)
Robust enhancers (2,034) with other ends	871 (42.8%)	940 (46.2%)	905 (44.4%)	876 (43.0%)	766 (37.7%)	1,618 (79.5%)
Comprehensive enhancers (2,284) with other ends	954 (41.8%)	1,037 (45.3%)	1,026 (44.9%)	999 (43.7%)	878 (38.4.%)	1,815 (79.5%)
Both: robust TCs with bait-ends and robust enhancers with OEs (6,233)	535 (8.5%)	592 (9.5%)	594 (9.5%)	512 (8.2%)	449 (7.2%)	1,142 (18.3%)
Robust TCs with bait-end and comprehensive enhancers with OE (6,389)	569 (8.9%)	657 (10.2%)	655 (10.2%)	573 (9.0%)	489 (7.7%)	1,259 (19.7%)
Are SNPs in enhancers (1 kb window) of interactions where both overlap: robust promoter with bait-end and robust enhancer with OE						
GWAS SNPs related to asthma (1,707)	0	1(*CD247*)	13 (*MPZL1,TIPRL,CD247*)	10 (*MPZL1,TIPRL,CD247*)	13 (*MPZL1,TIPRL,CD247*)	16 (*MPZL1,TIPRL,CD247*)
GWAS SNPs related to Childhood-onset asthma (492)	0	1(*CD247*)	3 (*MPZL1,TIPRL,CD247*)	3(*MPZL1,TIPRL,CD247*)	0	3 (*MPZL1,TIPRL,CD247*)
GWAS SNPs related to COPD (974)	9 (*GAB2*)	9(*GAB2*)	0	1	0	9(*GAB2*)
GWAS SNPs related to *response to bronchodilator* (2,524)	9(*GAB2*)	9(*GAB2*)	0	1	0	9(*GAB2*)
GWAS SNPs related to *forced expiratory volume* (FEV) (1,699)	3(*OBFC1,ITPRIP,CCDC147*)	3(*OBFC1,ITPRIP,CCDC147*)	3(*OBFC1,ITPRIP,CCDC147*)	3(*OBFC1,ITPRIP,CCDC147*)	3(*OBFC1,ITPRIP,CCDC147*)	3(*OBFC1,ITPRIP,CCDC147*)
GWAS SNPs related to *FEV/FEC ratio* (2,891)	1(*KCTD21*)	1(*KCTD21*)	1(*KCTD21*)	1(*KCTD21*)	1(*KCTD21*)	1(*KCTD21*)
Are SNPs in enhancers (10 kb window) of interactions where both overlap: robust promoter with bait-end and robust enhancer with OE						

GWAS SNPs related to asthma (1,707)	0	1 (*CD247*)	13 (*STXBP3, CD247,MPZL1,TIPRL, **)	10 (*STXBP3, CD247, MPZL1, TIPRL,**)	13 (*STXBP3, **)	16 (*STXBP3, CD247,MPZL1,TIPRL,**)
GWAS SNPs related to Childhood-onset asthma (492)	0	1(*CD247*)	3 (*CD247,MPZL1,TIPRL,**)	3 (*TIPRL*)	0	3 (*TIPRL,**)
GWAS SNPs related to COPD (974)	21 (*GAB2, PAK1*)	21 (*GAB2, PAK1*)	5 (*GALC,**)	10 (*DLG2,TMEM135,PICALM,GALC,**)	3 (*ZMIZ1,GALC,**)	26 (*ZMIZ1,DLG2,TMEM135,PICALM,GALC, **)
GWAS SNPs related to *response to bronchodilator* (2,524)	21 (*GAB2, PAK1)*	21 (*GAB2, PAK1*)	5 *(VAV3-AS1,WDR47,TRNAN-GUU,PRKCH, LOC101927780)*	10 *(VAV3-AS1,WDR47,TRNAN-GUU,PRKCH, LOC101927780)*	3 *(VAV3-AS1 ,WDR47,TRNAN-GUU)*	26 *(VAV3-AS1,WDR47, TRNAN-GUU,PRKCH,LOC101927780)*
GWAS SNPs related to forced expiratory volume (FEV) (1,699)	3 (*OBFC1,ITPRIP,CCDC147*)	3 (*OBFC1,ITPRIP,CCDC147*)	3 (*OBFC1,ITPRIP,CCDC147*)	3 (*OBFC1,ITPRIP,CCDC147)*	3 *(OBFC1,ITPRIP,CCDC147)*	3 *(OBFC1,ITPRIP,CCDC147)*
GWAS SNPs related to FEV/FEC ratio (2,891)	2 (*KCTD21,MXI1*)	1 *(KCTD21)*	18 *(VAV3-AS1,WDR47,TRNAN-GUU,KLLN,KCTD21,DUSP16,PRKCH,LOC101927780,*)*	2 *(VAV3-AS1,WDR47)*	2 *(VAV3-AS1,WDR47,)*	18 *(VAV3-AS1,WDR47, TRNAN-GUU,KLLN, KCTD21, DUSP16, PRKCH,LOC101927780,*)*

*Outside gene

Figure S14 | Expression of *RUNX3* across tissues

We looked at mRNA differential expressions in normal tissues according to GTEx [21] for the *RUNX3* gene. This gene is overexpressed mostly in the whole blood and lung.

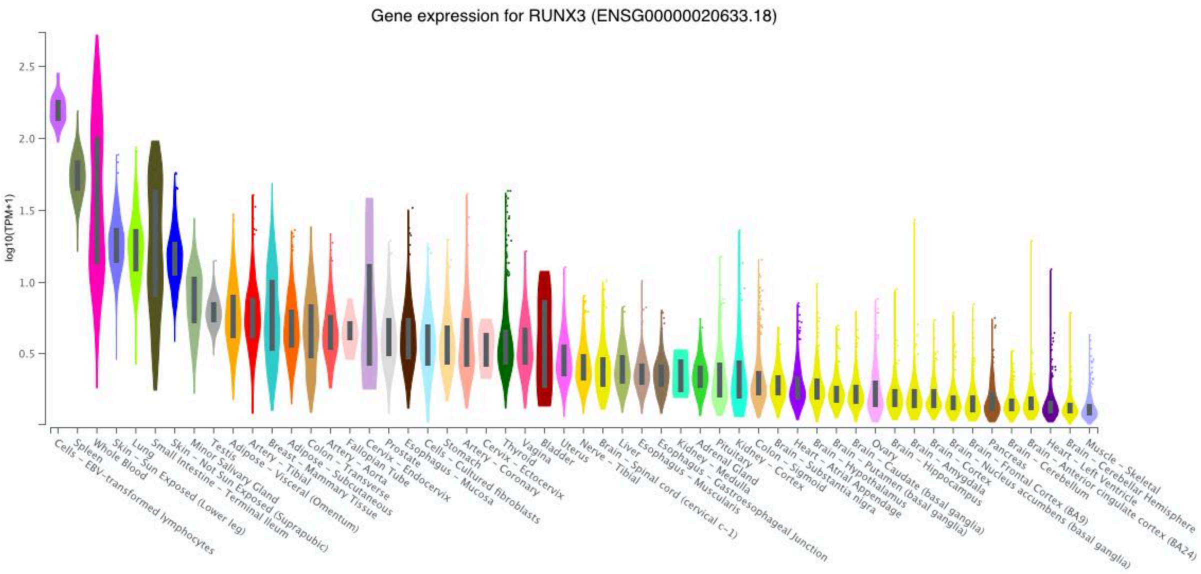

Figure S14: Expression of *RUNX3* across tissues

Table S15 | Enhancers coincide with McErlean et al.'s asthma enhancers

	McErlean (4,321)	
	0kb	*1kb*
Comprehensive enhancers (8,289)	103	146
Robust enhancers (4,738)	62	85
Novel comprehensive enhancers (3,277)	24	42
Novel robust enhancers (1,439)	12	22
Genomic enhancer clusters (1,492)	73	84
Comprehensive enhancers interacting with genes (1,636)	28	37
Robust enhancers interacting with genes (1,466)	22	28
Differentially expressed enhancers (100)	0	2 *(20 kb)*
Comparison with super enhancers and genomic region enhancers clusters		
	McErlean super enhancers (1,143)	
	0kb	*1kb*
Genomic enhancer clusters (1,492)	170	175

Table S15 : Identified enhancers are validated by McErlean asthma enhancers without flanking regions and with flanking regions of 1kb.

References

[1] H. Persson, A.T. Kwon, J.A. Ramilowski, G. Silberberg, C. Söderhäll, C. Orsmark-Pietras, B. Nordlund, J.R. Konradsen, M.J.L. de Hoon, E. Melén, Y. Hayashizaki, G. Hedlin, J. Kere, C.O. Daub, Transcriptome analysis of controlled and therapy-resistant childhood asthma reveals distinct gene expression profiles, J. Allergy Clin. Immunol. 136 (2015) 638–648.

[2] J.R. Konradsen, B. Nordlund, M. Lidegran, C. Pedroletti, H. Grönlund, M. van Hage, B. Dahlen, G. Hedlin, Swedish Network of Pediatric Allergists, Severe Asthma Network, Problematic severe asthma: a proposed approach to identifying children who are severely resistant to therapy, Pediatr. Allergy Immunol. 22 (2011) 9–18.

[3] M.C. Frith, E. Valen, A. Krogh, Y. Hayashizaki, P. Carninci, A. Sandelin, A code for transcription initiation in mammalian genomes, Genome Res. 18 (2008) 1–12.

[4] C. Nepal, Y. Hadzhiev, P. Balwierz, E. Tarifeño-Saldivia, R. Cardenas, J.W. Wragg, A.-M. Suzuki, P. Carninci, B. Peers, B. Lenhard, J.B. Andersen, F. Müller, Dual-initiation promoters with intertwined canonical and TCT/TOP transcription start sites diversify transcript processing, Nat. Commun. 11 (2020) 168.

[5] M.D. Robinson, D.J. McCarthy, G.K. Smyth, edgeR: a Bioconductor package for differential expression analysis of digital gene expression data, Bioinformatics 26 (2010) 139–140.

[6] E.Y. Chen, C.M. Tan, Y. Kou, Q. Duan, Z. Wang, G.V. Meirelles, N.R. Clark, A. Ma'ayan, Enrichr: interactive and collaborative HTML5 gene list enrichment analysis tool, BMC Bioinformatics 14 (2013) 128.

[7] A.R. Pico, T. Kelder, M.P. van Iersel, K. Hanspers, B.R. Conklin, C. Evelo, WikiPathways: Pathway Editing for the People, PLoS Biology 6 (2008) e184. https://doi.org/10.1371/journal.pbio.0060184.

[8] E. Tsitsiou, A.E. Williams, S.A. Moschos, K. Patel, C. Rossios, X. Jiang, O.-D. Adams, P. Macedo, R. Booton, D. Gibeon, K.F. Chung, M.A. Lindsay, Transcriptome analysis shows activation of circulating CD8+ T cells in patients with severe asthma, J. Allergy Clin. Immunol. 129 (2012) 95–103.

[9] FANTOM Consortium and the RIKEN PMI and CLST (DGT), A.R.R. Forrest, H. Kawaji, M. Rehli, J.K. Baillie, M.J.L. de Hoon, V. Haberle, T. Lassmann, I.V. Kulakovskiy, M. Lizio, M. Itoh, R. Andersson, C.J. Mungall, T.F. Meehan, S. Schmeier, N. Bertin, M. Jørgensen, E. Dimont, E. Arner, C. Schmidl, U. Schaefer, Y.A. Medvedeva, C. Plessy, M. Vitezic, J. Severin, C.A. Semple, Y. Ishizu, R.S. Young, M. Francescatto, I. Alam, D. Albanese, G.M. Altschuler, T. Arakawa, J.A.C. Archer, P. Arner, M. Babina, S. Rennie, P.J. Balwierz, A.G. Beckhouse, S. Pradhan-Bhatt, J.A. Blake, A. Blumenthal, B. Bodega, A. Bonetti, J. Briggs, F. Brombacher, A.M. Burroughs, A. Califano, C.V. Cannistraci, D. Carbajo, Y. Chen, M. Chierici, Y. Ciani, H.C. Clevers, E. Dalla, C.A. Davis, M. Detmar, A.D. Diehl, T. Dohi, F. Drabløs, A.S.B. Edge, M. Edinger, K. Ekwall, M. Endoh, H. Enomoto, M. Fagiolini, L. Fairbairn, H. Fang, M.C. Farach-Carson, G.J. Faulkner, A.V. Favorov, M.E. Fisher, M.C. Frith, R. Fujita, S. Fukuda, C. Furlanello, M. Furino, J.-I. Furusawa, T.B. Geijtenbeek, A.P. Gibson, T. Gingeras, D. Goldowitz, J. Gough, S. Guhl, R. Guler, S. Gustincich, T.J. Ha, M. Hamaguchi, M. Hara, M. Harbers, J. Harshbarger, A. Hasegawa, Y. Hasegawa, T. Hashimoto, M. Herlyn, K.J. Hitchens, S.J. Ho Sui, O.M. Hofmann, I. Hoof, F. Hori, L. Huminiecki, K. Iida, T. Ikawa, B.R. Jankovic, H. Jia, A. Joshi, G. Jurman, B. Kaczkowski, C. Kai, K. Kaida, A. Kaiho, K. Kajiyama, M. Kanamori-Katayama, A.S. Kasianov, T. Kasukawa, S. Katayama, S. Kato, S. Kawaguchi, H. Kawamoto, Y.I. Kawamura, T. Kawashima, J.S. Kempfle, T.J. Kenna, J.

Kere, L.M. Khachigian, T. Kitamura, S.P. Klinken, A.J. Knox, M. Kojima, S. Kojima, N. Kondo, H. Koseki, S. Koyasu, S. Krampitz, A. Kubosaki, A.T. Kwon, J.F.J. Laros, W. Lee, A. Lennartsson, K. Li, B. Lilje, L. Lipovich, A. Mackay-Sim, R.-I. Manabe, J.C. Mar, B. Marchand, A. Mathelier, N. Mejhert, A. Meynert, Y. Mizuno, D.A. de Lima Morais, H. Morikawa, M. Morimoto, K. Moro, E. Motakis, H. Motohashi, C.L. Mummery, M. Murata, S. Nagao-Sato, Y. Nakachi, F. Nakahara, T. Nakamura, Y. Nakamura, K. Nakazato, E. van Nimwegen, N. Ninomiya, H. Nishiyori, S. Noma, S. Noma, T. Noazaki, S. Ogishima, N. Ohkura, H. Ohimiya, H. Ohno, M. Ohshima, M. Okada-Hatakeyama, Y. Okazaki, V. Orlando, D.A. Ovchinnikov, A. Pain, R. Passier, M. Patrikakis, H. Persson, S. Piazza, J.G.D. Prendergast, O.J.L. Rackham, J.A. Ramilowski, M. Rashid, T. Ravasi, P. Rizzu, M. Roncador, S. Roy, M.B. Rye, E. Saijyo, A. Sajantila, A. Saka, S. Sakaguchi, M. Sakai, H. Sato, S. Savvi, A. Saxena, C. Schneider, E.A. Schultes, G.G. Schulze-Tanzil, A. Schwegmann, T. Sengstag, G. Sheng, H. Shimoji, Y. Shimoni, J.W. Shin, C. Simon, D. Sugiyama, T. Sugiyama, M. Suzuki, N. Suzuki, R.K. Swoboda, P.A.C. ’t Hoen, M. Tagami, N. Takahashi, J. Takai, H. Tanaka, H. Tatsukawa, Z. Tatum, M. Thompson, H. Toyodo, T. Toyoda, E. Valen, M. van de Wetering, L.M. van den Berg, R. Verado, D. Vijayan, I.E. Vorontsov, W.W. Wasserman, S. Watanabe, C.A. Wells, L.N. Winteringham, E. Wolvetang, E.J. Wood, Y. Yamaguchi, M. Yamamoto, M. Yoneda, Y. Yonekura, S. Yoshida, S.E. Zabierowski, P.G. Zhang, X. Zhao, S. Zucchelli, K.M. Summers, H. Suzuki, C.O. Daub, J. Kawai, P. Heutink, W. Hide, T.C. Freeman, B. Lenhard, V.B. Bajic, M.S. Taylor, V.J. Makeev, A. Sandelin, D.A. Hume, P. Carninci, Y. Hayashizaki, A promoter-level mammalian expression atlas, Nature 507 (2014) 462–470.

[10] M. Boyd, M. Thodberg, M. Vitezic, J. Bornholdt, K. Vitting-Seerup, Y. Chen, M. Coskun, Y. Li, B.Z.S. Lo, P. Klausen, P. Jan Schweiger, A.G. Pedersen, N. Rapin, K. Skovgaard, K. Dahlgaard, R. Andersson, T.B. Terkelsen, B. Lilje, J.T. Troelsen, A.M. Petersen, K.B. Jensen, I. Gögenur, P. Thielsen, J.B. Seidelin, O.H. Nielsen, J.T. Bjerrum, A. Sandelin, Characterization of the enhancer and promoter landscape of inflammatory bowel disease from human colon biopsies, Nat. Commun. 9 (2018) 1661.

[11] Index of /5/datafiles/latest/extra/enhancers, (n.d.). https://fantom.gsc.riken.jp/5/datafiles/latest/extra/Enhancers/ (accessed May 21, 2021).

[12] K.S. Pollard, M.J. Hubisz, K.R. Rosenbloom, A. Siepel, Detection of nonneutral substitution rates on mammalian phylogenies, Genome Res. 20 (2010) 110–121.

[13] A. Siepel, Evolutionarily conserved elements in vertebrate, insect, worm, and yeast genomes, Genome Research 15 (2005) 1034–1050. https://doi.org/10.1101/gr.3715005.

[14] F. Ramírez, D.P. Ryan, B. Grüning, V. Bhardwaj, F. Kilpert, A.S. Richter, S. Heyne, F. Dündar, T. Manke, deepTools2: a next generation web server for deep-sequencing data analysis, Nucleic Acids Res. 44 (2016) W160–5.

[15] G. Liu, M.A. Cooley, P.M. Nair, C. Donovan, A.C. Hsu, A.G. Jarnicki, T.J. Haw, N.G. Hansbro, Q. Ge, A.C. Brown, H. Tay, P.S. Foster, P.A. Wark, J.C. Horvat, J.E. Bourke, C.L. Grainge, W.S. Argraves, B.G. Oliver, D.A. Knight, J.K. Burgess, P.M. Hansbro, Airway remodelling and inflammation in asthma are dependent on the extracellular matrix protein fibulin-1c, J. Pathol. 243 (2017) 510–523.

[16] I. Mahmutovic Persson, M. Menzel, S. Ramu, S. Cerps, H. Akbarshahi, L. Uller, IL-1β mediates lung neutrophilia and IL-33 expression in a mouse model of viral-induced asthma exacerbation, Respir. Res. 19 (2018) 16.

[17] A. Kloetgen, P. Thandapani, P. Ntziachristos, Y. Ghebrechristos, S. Nomikou, C. Lazaris, X. Chen, H. Hu, S. Bakogianni, J. Wang, Y. Fu, F. Boccalatte, H. Zhong, E. Paietta, T. Trimarchi, Y. Zhu, P. Van Vlierberghe, G.G. Inghirami, T. Lionnet, I. Aifantis, A. Tsirigos, Three-dimensional chromatin landscapes in T cell acute lymphoblastic leukemia, Nat. Genet. 52 (2020) 388–400.

[18] Feng Yue lab at northwestern university, (n.d.). http://yuelab.org/ (accessed May 17,

2021).
[19] B.M. Javierre, O.S. Burren, S.P. Wilder, R. Kreuzhuber, S.M. Hill, S. Sewitz, J. Cairns, S.W. Wingett, C. Várnai, M.J. Thiecke, F. Burden, S. Farrow, A.J. Cutler, K. Rehnström, K. Downes, L. Grassi, M. Kostadima, P. Freire-Pritchett, F. Wang, BLUEPRINT Consortium, H.G. Stunnenberg, J.A. Todd, D.R. Zerbino, O. Stegle, W.H. Ouwehand, M. Frontini, C. Wallace, M. Spivakov, P. Fraser, Lineage-Specific Genome Architecture Links Enhancers and Non-coding Disease Variants to Target Gene Promoters, Cell 167 (2016) 1369–1384.e19.
[20] K.K. Dey, S. Gazal, B. van de Geijn, S.S. Kim, J. Nasser, J.M. Engreitz, A.L. Price, SNP-to-gene linking strategies reveal contributions of enhancer-related and candidate master-regulator genes to autoimmune disease, Cell Genom 2 (2022). https://doi.org/10.1016/j.xgen.2022.100145.
[21] GTEx Portal, (n.d.). https://www.gtexportal.org/home/ (accessed April 15, 2021).
